# BRN2 suppresses apoptosis, reprograms DNA damage repair, and is associated with a high somatic mutation burden in melanoma

**DOI:** 10.1101/gad.314633.118

**Published:** 2019-03-01

**Authors:** Katharine Herbert, Romuald Binet, Jean-Philippe Lambert, Pakavarin Louphrasitthiphol, Halime Kalkavan, Laura Sesma-Sanz, Carla Daniela Robles-Espinoza, Sovan Sarkar, Eda Suer, Sarah Andrews, Jagat Chauhan, Nicola D. Roberts, Mark R. Middleton, Anne-Claude Gingras, Jean-Yves Masson, Lionel Larue, Paola Falletta, Colin R. Goding

**Affiliations:** 1 Ludwig Institute for Cancer Research, Nuffield Department of Clinical Medicine, University of Oxford, Headington, Oxford OX3 7DQ, United Kingdom;; 2 Lunenfeld-Tanenbaum Research Institute, Mount Sinai Hospital, Toronto, Ontario M5G 1X5, Canada;; 3 Department of Molecular Medicine, Cancer Research Centre, Université Laval, Quebec G1V 0A6, Canada; CHU de Québec Research Center, CHUL, Quebec G1V 4G2, Canada;; 4 Department of Immunology, St. Jude Children's Research Hospital, Memphis, Tennessee 38105, USA;; 5 Genome Stability Laboratory, CHU de Oncology Division, Québec Research Center, Québec City, Quebec G1R 3S3, Canada;; 6 Department of Molecular Biology, Medical Biochemistry, and Pathology, Laval University Cancer Research Center, Québec City, Quebec G1V 0A6, Canada;; 7 Laboratorio Internacional de Investigación Sobre el Genoma Humano, Universidad Nacional Autónoma de México, Santiago de Querétaro 76230, Mexico;; 8 Experimental Cancer Genetics, The Wellcome Trust Sanger Institute, Hinxton, Cambridgeshire CB10 1SA, United Kingdom;; 9 Department of Oncology, University of Oxford, Headington, Oxford OX3 7DQ, United Kingdom;; 10 The Cancer Genome Project, The Wellcome Trust Sanger Institute, Hinxton, Cambridgeshire CB10 1SA, United Kingdom;; 11 Department of Molecular Genetics, University of Toronto, Toronto, Ontario M5S 1A8, Canada;; 12 Institut Curie, PSL Research University, Normal and Pathological Development of Melanocytes, U1021, Institut National de la Santé et de la Recherche Médicale (INSERM), 91405 Orsay, France;; 13 University Paris-Sud, University Paris-Saclay, UMR 3347, Centre National de la Recherche Scientifique (CNRS), 91505 Orsay, France;; 14 Equipe Labellisée Ligue Contre le Cancer, 91405 Orsay, France;; 15 Università Vita-Salute San Raffaele, Milano, 20132 Milano MI, Italy

**Keywords:** BRN2, POU3F2, Ku80, melanoma, somatic mutation burden, BCL2, apoptosis, nonhomologous end-joining

## Abstract

Whether cell types exposed to a high level of environmental insults possess cell type-specific prosurvival mechanisms or enhanced DNA damage repair capacity is not well understood. BRN2 is a tissue-restricted POU domain transcription factor implicated in neural development and several cancers. In melanoma, BRN2 plays a key role in promoting invasion and regulating proliferation. Here we found, surprisingly, that rather than interacting with transcription cofactors, BRN2 is instead associated with DNA damage response proteins and directly binds PARP1 and Ku70/Ku80. Rapid PARP1-dependent BRN2 association with sites of DNA damage facilitates recruitment of Ku80 and reprograms DNA damage repair by promoting Ku-dependent nonhomologous end-joining (NHEJ) at the expense of homologous recombination. BRN2 also suppresses an apoptosis-associated gene expression program to protect against UVB-, chemotherapy- and vemurafenib-induced apoptosis. Remarkably, BRN2 expression also correlates with a high single-nucleotide variation prevalence in human melanomas. By promoting error-prone DNA damage repair via NHEJ and suppressing apoptosis of damaged cells, our results suggest that BRN2 contributes to the generation of melanomas with a high mutation burden. Our findings highlight a novel role for a key transcription factor in reprogramming DNA damage repair and suggest that BRN2 may impact the response to DNA-damaging agents in BRN2-expressing cancers.

## Abstract (precis)

Herbert et al. show that BRN2 is associated with DNA damage response proteins and suppresses an apoptosis-associated gene expression program to protect against UVB-, chemotherapy-, and vemurafenib-induced apoptosis.

Exposure of the skin to solar UV irradiation can lead to accumulation of unrepaired DNA damage. While this is not a major issue for short-lived keratinocytes, for long-lived cells at the basal layer of the epidermis accumulation of DNA damage can lead to malignant transformation. This is reflected in the frequently very high mutational burden of cutaneous melanoma (Hodis et al. 2012; Krauthammer et al. 2012; Alexandrov et al. 2013), a highly aggressive skin cancer originating from melanocytes in the skin. Once cells begin proliferating as a consequence of senescence bypass combined with acquisition of an activated oncogene such as BRAF (Shain and Bastian 2016), a high mutational load may impose a selective pressure for cells bearing enhanced resistance to cell death as well as providing an increased probability of therapy resistance. Whether cutaneous melanomas possess cell type-specific prosurvival mechanisms or enhanced DNA damage repair (DDR) capacity is not well understood.

Lineage identity is determined by the activity of tissue-restricted transcription factor binding to sequence elements within genes associated with cell type-specific functions (Long et al. 2016). Given their tissue-restricted expression, lineage-determining transcription factors represent candidates for imposition of cell type-specific responses to DNA damage. Consistent with this, in melanocytes the lineage-determining microphthalmia-associated transcription factor (MITF) that controls melanoma differentiation, proliferation, and invasion (Hoek and Goding 2010; Kawakami and Fisher 2017) can regulate expression of DDR genes (Strub et al. 2011).

The highly conserved POU domain family members are key regulators of development, particularly stem cell identity, self-renewal, reprogramming, and cell fate determination (Ryan and Rosenfeld 1997; Veenstra et al. 1997; Takahashi and Yamanaka 2006). Among the POU domain proteins, BRN2 (POU3F2) is critical for correct cortical neuronal spatiotemporal organization and neuronal precursor survival (Fujii and Hamada 1993; Sugitani et al. 2002), has been implicated in direct reprogramming of fibroblasts to neurons (Ambasudhan et al. 2011) and can convert astrocytes into neurons (Zhu et al. 2018). BRN2 is required for development of specific neuroendocrine cell lineages in the hypothalamus (Nakai et al. 1995; Schonemann et al. 1995) and is also expressed in other precursor cells of neural crest origin (Andersen and Rosenfeld 2001). Intriguingly, BRN2 is overexpressed in tumors with neuroendocrine cell origin such as glioblastoma (Schreiber et al. 1990) and small-cell and carcinoid lung cancer (Ishii et al. 2013) and is a key driver of neuroendocrine prostate cancer proliferation (Bishop et al. 2017). Although absent or at low levels in melanoblasts and differentiated melanocytes in vivo (Goodall et al. 2004a), BRN2 re-emerges as a critical driver of invasiveness and regulator of proliferation during melanomagenesis (Eisen et al. 1995; Cook and Sturm 2008; Goodall et al. 2008; Besch and Berking 2014; Zeng et al. 2018; Fane et al. 2019), where it is up-regulated by melanoma-associated signaling downstream from BRAF (Goodall et al. 2004b), β-catenin (Goodall et al. 2004a), or PI3K (Bonvin et al. 2012), as well as by E2F1 (Zeng et al. 2018). Importantly, BRN2 may contribute to melanoma progression through regulation of MITF expression, repressing or activating the *MITF* promoter depending on cellular context (Goodall et al. 2008; Wellbrock et al. 2008). In vivo (Goodall et al. 2008) or in 3D culture (Thurber et al. 2011), MITF and BRN2 are expressed in distinct subpopulations of melanoma cells, likely reflecting a feedback loop in which MITF activates miR-211 expression that represses BRN2 to alleviate the suppression of MITF (Boyle et al. 2011). BRN2 is also required for outgrowth of melanoma metastases in mouse xenografts (Simmons et al. 2017) and can epigenetically reprogram melanoma cells via up-regulation of the H3K27 methyl transferase EZH2 (Fane et al. 2017). Moreover, BRN2 expression increases as melanomas progress to become invasive, consistent with BRN2 in vivo being expressed specifically in migrating melanoma cells within tumors (Goodall et al. 2008; Pinner et al. 2009) and promoting melanoma invasion in vitro and in vivo (Arozarena et al. 2011; Thurber et al. 2011; Fane et al. 2017; Zeng et al. 2018). Given the key role played by BRN2 as a tissue-restricted transcription factor expressed in melanoma but not in other cells in the skin (Richmond-Sinclair et al. 2008; Zeng et al. 2018), we aimed here to determine whether in addition to contributing to melanoma progression, BRN2 might also contribute to protecting cells from the consequences of DNA damage.

## Results

### BRN2 interacts with DDR factors via its DNA-binding domain

The POU domain transcription factor BRN2 plays a critical role in development and a range of cancers. In melanoma BRN2 regulates proliferation (Goodall et al. 2004a) and promotes invasion (Goodall et al. 2008; Arozarena et al. 2011; Thurber et al. 2011; Fane et al. 2017; Zeng et al. 2018). This is reflected in the correlation between BRN2 expression in The Cancer Genome Atlas (TCGA) melanoma cohort and the well-characterized melanoma-associated Verfaillie (Verfaillie et al. 2015) invasive gene expression signature, whereas BRN2 is anticorrelated with the Verfaillie proliferative gene expression signature (Supplemental Fig. S1A). However, remarkably little is known about how BRN2 exerts its effects. To establish what cofactors might be mediating its function we used affinity purification coupled to mass spectrometry (AP-MS) to perform an unbiased search for BRN2 interactors. Preliminary analysis indicated that efficient immunoprecipitation of endogenous BRN2 was not readily achievable using currently available anti-BRN2 antibodies. We therefore used human 501mel melanoma cells that endogenously express BRN2 to generate a cell line expressing stable, doxycycline-inducible Flag epitope-tagged BRN2 (Supplemental Fig. S1B). This allowed controlled expression of BRN2 protein and ensured a high specificity of immunoprecipitation of the Flag-tagged BRN2 protein, which was followed by AP-MS analysis.

**Figure 1.**
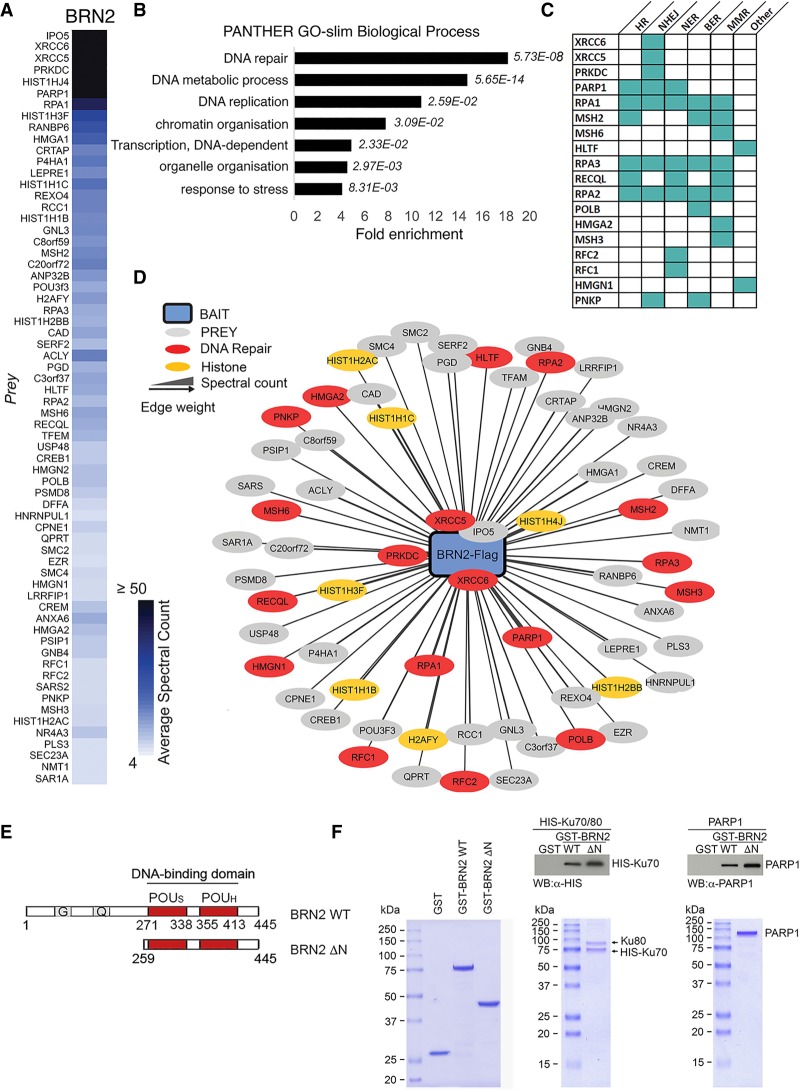
BRN2 binds proteins involved in the DNA damage response. (*A*) Heat map representing average spectral counts of proteins identified to copurify with BRN2 by AP-MS after SAINTexpress analysis. False discovery rate cutoff of ≤1%. (*B*) Gene ontology analysis of BRN2-binding partners. PANTHER GO-slim biological processes with greater than fourfold enrichment on overrepresentation analysis. Binomial test *P*-values in italics. (*C*) Table of DDR pathways in which BRN2 binding partners are involved. (HR) Homologous recombination; (NHEJ) nonhomologous end joining; (NER) nucleotide excision repair; (BER) base excision repair; (MMR) mismatch repair. (*D*) BRN2 binding partners arranged by Cytoscape edge-weighted spring-embedded layout, where the summed spectral count is inversely proportional to a prey distance from BRN2. GO annotation: DNA repair (red), Histones (yellow), and other (gray). (*E*) Diagram depicting BRN2 wild-type and N-terminal deletion mutant used in pull-down assays. Numbers indicate amino acid residues. The POU domain ([POU_S_] POU-specific domain; [POU_H_] POU homeodomain) is shown in red, and the glycine-rich (G) and glutamine-rich (Q) regions are indicated. (*F*) GST-pull down assays using purified bacterially expressed GST-BRN2 wild type together with purified recombinant HIS-tagged Ku70/Ku80 complex or PARP1. The purified proteins used are shown in the indicated Coomassie-stained gels. After pull-down using glutathione beads, samples were Western blotted for HIS-tagged Ku70 or PARP1 as indicated.

We initially undertook the AP-MS analysis using cells in which ectopic BRN2 was not induced by doxycycline since this basal level of ectopic BRN2-Flag was around fourfold to fivefold higher than endogenous BRN2 expressed in 501mel cells (Supplemental Fig. S1C), a similar level to that expressed in Lu1205 (Bonvin et al. 2012) or A375M (Goodall et al. 2004a) melanoma cell lines. However, in these experiments we did not detect the expected transcription cofactors, but instead found several DDR factors copurifying with BRN2, including DNA-dependent protein kinase (DNAPK and PRKDC), Ku70 (XRCC6), and Ku80 (XRCC5) as well as importin 5 (IPO5). Given the role of BRN2 in regulating transcription this was surprising. We therefore repeated the AP-MS analysis using 10 ng of doxycycline to increase the levels of BRN2-Flag and the robustness of the purification. Using SAINTexpress (significance analysis of interactome), we identified interaction partners found to be statistically enriched with Flag-tagged BRN2 versus our untagged control purifications. Using a threshold of false discovery rate of ≤1%, 66 proteins were identified as significant copurifying factors of BRN2 (Fig. 1A). Again, no transcription cofactors copurified with BRN2, such as p300 that was previously described as binding BRN2 (Smit et al. 2000). However, consistent with the preliminary AP-MS analysis using uninduced ectopic expression of BRN2, gene ontology overrepresentation analysis using PANTHER revealed that the most overrepresented PANTHER GO-slim biological process was DNA repair with >18-fold enrichment compared with reference genomes (*P* = 5.73 × 10^8^) (Fig. 1B). The interaction of BRN2 with the DDR factor Ku80, identified by the MS approach as one of the highest abundance BRN2 interacting factors, was validated by coimmunoprecipitation followed by Western blotting (Supplemental Fig. S1D). These data raised the possibility that BRN2 may have a function in DDR outside of its canonical role as a transcription factor.

BRN2 has a highly disordered N-terminal region, notable for polyglutamine and polyglycine tracts but with no established protein domains, a bipartite DNA-binding domain comprising the POU-specific domain (POU_S_) and POU-homeodomain (POU_H_), and a short C-terminal region (Supplemental Fig. S1E). To establish which part of BRN2 was bound by the copurifying factors identified, we created doxycycline-inducible cell lines expressing truncation mutants consisting of the conserved POU domain plus the C-terminal region (ΔN), a version lacking the C-terminal region (ΔC), or the N-terminal region alone (N-term) (Supplemental Fig. S1E). The inducibility, size, and subcellular localization of these mutants were verified by Western blotting (Supplemental Fig. S1F) and immunofluorescence (Supplemental Fig. S1G). Since the POU domain contains a highly conserved nuclear localization signal (NLS) (Sock et al. 1996), all proteins were nuclear except the N-terminal region that lacks an NLS. We next used 10 ng of doxycycline to induce the expression of BRN2 wild-type and mutants to similar levels (Supplemental Fig. S2A). Immunoprecipitation of the ectopically expressed proteins using anti-Flag followed by MS of the immunoprecipitated BRN2-associated proteins identified significant binding to BRN2 by a further 79 proteins (Supplemental Fig. S2B). Again, DDR proteins were highly enriched and no transcriptional cofactors were identified. Differential recovery of interacting factors between the mutants indicates that the association with DDR proteins is highly specific and is mediated via the highly conserved POU DNA-binding domain (Supplemental Fig. S2B).

DNA lesions range from damage to individual bases through to double-strand breaks (DSBs), with different types of lesion having dedicated repair pathways, although many of the DDR proteins play roles in multiple repair pathways. Analysis of the proteins copurifying with BRN2 indicated that they are involved in many DDR processes with no pathway predominant (Fig. 1C). Notably, the function of many of the BRN2-interacting DDR proteins identified is in the initiation of the DDR—specifically chromatin remodeling (e.g., poly-ADP-ribose polymerase [PARP]) (Dantzer et al. 2006)—or marking DNA damage and recruitment of DDR pathway components such as the DNAPK complex consisting of DNAPK_CS_ (PRKDC), Ku80 (XRCC5), and Ku70 (XRCC6) (Blackford and Jackson 2017). This suggested that BRN2 may be involved in the early phases of DNA damage recognition and response rather than in a specific repair pathway. The 18 proteins implicated in DNA repair and a further seven histone proteins that copurify with BRN2 wild type weighted by average spectral count are shown in Figure 1D. This demonstrates that except for IPO5, presumably involved in BRN2 nuclear import, the highest abundance factors copurifying with BRN2 are DNA repair proteins or histones.

Given the number of proteins identified in the AP-MS analysis, it seemed likely that many would interact with BRN2 indirectly, for example, via common chromatin interactions. Therefore, to verify that the most significant interactors bound BRN2 directly, we bacterially expressed and purified GST-BRN2 wild type and the ΔN mutant, which retains the POU domain that the AP-MS data highlighted was sufficient for interaction with the majority of copurifying proteins (Fig. 1E), and examined their interaction with recombinant purified Ku70/Ku80 dimers and PARP1. The results (Fig. 1F) revealed that purified full-length BRN2 was able to bind both PARP1 and the Ku70/Ku80 dimer. Consistent with the AP-MS data, the ΔN mutant exhibited a moderately increased ability to bind these proteins.

### Inhibition of BRN2 function by the N-terminal domain

**Figure 2.**
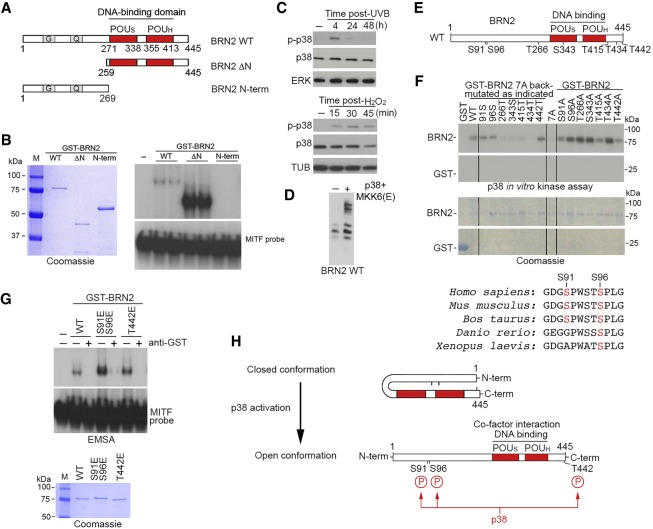
The N-terminal region of BRN2 inhibits DNA binding and can be phosphorylated by p38. (*A*) Diagram depicting BRN2 wild type and deletion mutants used DNA-binding assays. Numbers indicate amino acid residues. The POU domain is shown in red. (*B*) In vitro DNA-binding (EMSA) assay in duplicate using a radiolabeled MITF promoter probe and bacterially expressed and purified GST-BRN2 wild-type, ΔN mutant, or N-terminal region (amino acids 1–269). The Coomassie-stained gel (*left*) shows the purified proteins used, with the EMSA shown at the *right*. The unbound probe (*bottom*) and bound probe (*top*) are indicated. (*C*) Western blot using indicated antibodies of 501mel cells exposed to 150 J/m^2^ UVB (*top*) or 2.5 mM H_2_O_2_ (*bottom*) for the indicated times. (*D*) Western blot showing relative migration of BRN2 wild type transiently expressed in 501mel cells with or without cotransfected p38 and constitutively active MKK6(E) expression vectors. Samples were analyzed by SDS PAGE using a gel containing 50 µM Phos-tag reagent to efficiently separate phosphorylated forms. (*E*) Schematic showing wild-type BRN2 in which the POU_S_ and POU_H_ are indicated in red. The *top* numbers indicate amino acids at the N and C termini of BRN2, and the *bottom* numbers indicate the positions of S/TP motifs. (*F*) In vitro kinase assay using purified p38 and indicated wild-type and mutated GST-BRN2 fusion proteins. The in vitro kinase assay is shown in the *top* panel, and the Coomassie-stained purified BRN2 protein is shown in the *bottom* panel. The *top* and *bottom* parts of the kinase assay and Coomassie gel were run on the same gel but have been cropped to save space. An alignment of BRN2 showing amino acid conservation between species in the vicinity of S91 and S96 is shown *below*. (*G*) In vitro DNA-binding (EMSA) assay using a radiolabeled MITF promoter probe and bacterially expressed and purified GST-BRN2 wild-type or indicated mutants. The Coomassie-stained gel (bottom) shows the purified proteins used, with the EMSA shown above. The unbound probe (*bottom*) and bound probe (*top*) are indicated. Anti-GST antibody was used to confirm that the bound probe was recognized by GST-BRN2. (*H*) Model to explain the potential role of phosphorylation of the BRN2 N-terminal region. In the absence of phosphorylation on S91 and/or S96 BRN2 is in a closed conformation in which the N-terminal domain masks the POU domain, restricting DNA binding and interaction with cofactors. Phosphorylation of the N-terminal residues inhibits the intramolecular interaction to expose the POU domain, thereby enabling BRN2 to bind DNA and interact better with its cofactors.

The enhanced binding of interacting factors to BRN2 lacking its N-terminal region observed in the direct interaction assays (Fig. 1F) as well as in the AP-MS approach (Supplemental Fig. S2B) led us to hypothesize that the ability of BRN2 to interact with its cofactors was via its conserved POU DNA-binding domain and was inhibited by the N-terminal region. If so, deletion of the N-terminal region should enhance DNA binding. To test this, we used the bacterially expressed and purified BRN2 full-length and ΔN mutant (Fig. 2A) in an in vitro electrophoretic mobility shift assay (EMSA) using a radiolabeled probe containing a BRN2-binding site previously identified in the MITF promoter (Goodall et al. 2008). The non-DNA-binding N-terminal region (amino acids 1–269) was used as a negative control. The results (Fig. 2B) indicated that for the same amount of input protein, the ΔN mutant had substantially increased capacity to bind DNA compared with full-length BRN2. No DNA binding was detected using the purified recombinant N-terminal region. These data are consistent with the N-terminal domain of BRN2 interfering with the ability of BRN2 to bind DNA, and also limiting its capacity to associate via the POU domain with its DDR-related cofactors. Since DNA binding is a prerequisite for sequence-specific regulation of gene expression, it also suggested that the N-terminal domain might also suppress BRN2's capacity to control gene expression.

Collectively, these observations raised the possibility that the ability of the N-terminal region to inhibit BRN2 DNA binding would be regulated by signals associated with DNA damage. UVB irradiation, responsible for much of the mutation burden in melanoma, can activate a signaling cascade culminating in activation of the p38 stress-activated kinase (Son et al. 2013). This is exemplified by increased phosphorylated (activated) p38 in cells following UVB exposure or treatment with H_2_O_2_ that generates ROS (Fig. 2C). Initial experiments using a Phos-tag gel that separates phosphorylated forms of proteins by SDS PAGE (Fig. 2D) revealed that coexpression of wild-type BRN2 together with p38 and its upstream activating kinase MKK6 induced a mobility shift in BRN2, indicating that BRN2 can be phosphorylated in response to activation of p38 signaling. p38 phosphorylates serine or threonine residues immediately N-terminal to a proline (S/TP motifs). Examination of BRN2's amino acid sequence revealed seven candidate target sites (Fig. 2E). To map the potential p38 phosphorylation sites, we performed in vitro kinase assays using p38 and bacterially expressed and purified wild-type and mutant BRN2 in which all seven S/TP motifs were mutated to alanine (7A) or back-mutated one at a time to serine or threonine. The results (Fig. 2F, left) indicated that while the 7A mutant lacking all potential p38 target sites was not phosphorylated by p38, phosphorylation was detected when S91, S96, or T442 were present. Reduced phosphorylation of BRN2 was also observed if S91 or T442 were individually mutated to alanine (Fig. 2F, right), as well as with the T415A mutant. However, as the presence of T415 did not increase phosphorylation in the 7A background we did not pursue this potential modification site further. Together, these data suggest that BRN2 can be phosphorylated on three residues by p38, two of which (S91 and S96) lie within the N-terminal region that inhibits both DNA binding (Fig. 2B) and interaction with cofactors (Supplemental Fig. S2B). Notably, S91 and especially S96 are evolutionarily conserved within BRN2 from different species (Fig. 2F, bottom panel). Glutamic acid substitution of S91 and S96 or T442 to partially mimic phosphorylation led to increased DNA binding to a well-characterized BRN2 target site from the MITF promoter in vitro using an EMSA assay (Fig. 2G). Note that phosphorylation of BRN2 on T442 has been detected in high-throughput phospho-proteomic studies (Hornbeck et al. 2019). Although these analyses detected a number of C-terminal phosphorylation sites, none found any post-translational modifications or peptides derived from BRN2 N-terminal to the POU domain. Indeed, our own AP-MS analysis of BRN2 failed to detect any peptides derived from the N-terminal region of BRN2 due to this glycine-rich region of the protein being refractory to MS analysis. Nevertheless, our data are consistent with a model (Fig. 2H) in which phosphorylation of BRN2 on S91 and S96 by p38, or potentially, other serine/proline kinases, including cyclin-dependent kinases, may unmask the BRN2 POU domain, leading to increased DNA binding and better association with the DDR proteins detected in the AP-MS.

### PARP-dependent recruitment of BRN2 to sites of DNA damage

**Figure 3.**
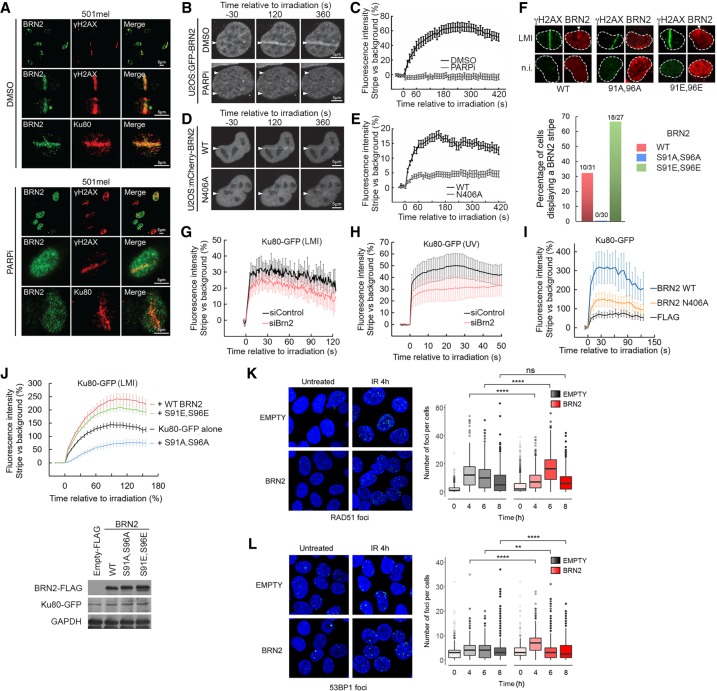
BRN2 facilitates Ku recruitment to sites of DNA damage. (*A*) Immunofluorescence of 501mel cells 90 sec after LMI. Cells were stained with antibodies against γH2AX or Ku80 and BRN2. Cells were pretreated with DMSO (*top* panels) or 10 µM PARP inhibitor olaparib (*bottom* panels) for 3 h prior to irradiation. (*B*) Still images from live cell imaging of U-2 OS cells transiently transfected with GFP-BRN2 wild-type expression vector with or without PARP inhibitor treatment as above and subject to LMI as in *A*. (*C*) Quantification of live imaging shown in *B*. Data shown are the mean fluorescence intensity change of irradiated stripe versus background per cell expressed as mean ± SEM. DMSO, *N* = 11; PARPi, *N* = 15. (*D*) Still images from live cell imaging of U-2 OS cells transiently transfected with mCherry-BRN2 wild type or mCherry-BRN2 N406A expression vectors and subject to LMI. (*E*) Quantification of live imaging shown in *D*. Data shown are the mean fluorescence intensity change of irradiated stripe versus background per cell expressed as mean ± SEM. BRN2 wild type, *N* = 27; BRN2 N406A, *N* = 52. (*F*) Immunofluorescence images using anti-Flag or anti-γH2AX antibodies of U-2 OS cells expressing Flag-BRN2 wild-type or indicated mutants in cells after LMI or in nonirradiated (n.i.) cells. Quantification (*below*) of numbers of LMI treated cells with BRN2 colocalizing with the γH2AX stripe. (*G*,*H*) Results of live-cell imaging LMI of 501mel cells transfected with Ku80-GFP and depleted for BRN2 after LMI (*G*) or UVB microirradiation (*H*). Data shown are the mean fluorescence intensity change of irradiated stripe versus background per cell expressed as mean ± SEM. LMI: siControl, *N* = 8; siBRN2, *N* = 9. UV: siNT, *N* = 11; siBRN2 *N* = 7. (*I*,*J*) Results of live cell imaging of U-2 OS cells transfected with Ku80-GFP alone or together with indicated BRN2 expression vectors. The *bottom* panel in *J* is Western blot showing relative expression levels of indicated BRN2 and Ku80-GFP proteins. Data shown are the mean fluorescence intensity change of irradiated stripe versus background per cell expressed as mean ± SEM. (*I*) Flag *N* = 15; BRN2 wild type, *N* = 12; BRN2 N406A, *N* = 12. (*J*) Ku80-GFP alone, *N* = 196; +wild-type BRN2, *N* = 167; +S91E, S96E, *N* = 151; +S91A, S96A, *N* = 196. (*K*,*L*) Control U-2 OS cells or cells expressing BRN2 were treated with 2 Gy γ-irradiation to induce DSBs before being subject to immunofluorescence with anti-RAD51 (*K*) or anti-53BP1 (*L*) antibodies. Representative images from a 4-h time point are shown. Quantification is presented as foci per cell over time. (**) *P* < 0.01; (****) *P* < 0.0001. Analysis by unpaired Student's *t*-test.

Having determined that BRN2 is associated with DDR proteins, particularly those involved in recognition and onset of the DDR, we wanted to establish whether BRN2 could be recruited to sites of DNA damage. Laser microirradiation (LMI) followed by immunofluorescence revealed that endogenous BRN2 is recruited to sites of DNA damage; 90 sec after LMI, BRN2 had accumulated at the damaged area together with the two well-established DNA damage markers γH2AX and Ku80 (Fig. 3A, top panels). PARP, one of the early DNA damage response proteins, is required for recruitment of a specific subset of DDR proteins to sites of DNA damage (Dantzer et al. 2006; Gupte et al. 2017). PARP1, the most highly expressed PARP family member in melanoma based on single-cell sequencing data (Supplemental Fig. S3A; Tirosh et al. 2016) was also one of the most highly enriched binding partners of BRN2 (Fig. 1A,D; Supplemental Fig. S2B). Notably, the PARP inhibitor olaparib prevented BRN2 recruitment to sites of damage, whereas the inhibitor had little effect on the γH2AX signal or Ku80 recruitment (Fig. 3A, bottom panels). Consistent with the results obtained with olaparib, recruitment of endogenous BRN2 to sites of damage was also reduced by siRNA-mediated depletion of PARP1 (Supplemental Fig. S3B).

The recruitment of endogenous BRN2 to sites of DNA damage marked by colocalization with γH2AX occurred within 5 min after LMI. To obtain a more precise analysis of the dynamics of BRN2 recruitment to sites of DNA damage we performed live cell imaging of GFP-BRN2 expressed in U-2 OS osteosarcoma cells that are frequently used as a model system for examination of DNA repair (Kochan et al. 2017) and which do not express endogenous BRN2. The results (Fig. 3B,C; Supplemental Movies S1,S2) revealed that BRN2 recruitment to sites of DNA damage is rapid, and peaks by 4 min following LMI, with no GFP-BRN2 detectable at sites of LMI damage in cells pretreated with PARP inhibitor. Similar results were obtained using siRNA-mediated depletion of PARP1 (Supplemental Fig. S3C). Recruitment of BRN2 to LMI-induced DNA damage was recapitulated using UV to micro-irradiate cells where BRN2 was recruited to damage (Supplemental Fig. S3D), with recruitment being diminished, though not prevented, by olaparib or siRNA-mediated PARP depletion. Using UVB to irradiate cells led to a minor, though not significant, increase in reactive oxygen species (ROS) that was increased significantly by BRN2 depletion, with the increase prevented by addition of the ROS scavenger N-acetyl cysteine (NAC) (Supplemental Fig. S3E). Although LMI can generate ROS, pretreatment of cells with NAC did not prevent BRN2 recruitment to LMI-induced DNA damage and, if anything, promoted a moderate increase in BRN2 association with damage (Supplemental Fig. S3F).

To establish whether DNA binding is required for BRN2 recruitment to sites of DNA damage, we generated a non-DNA-binding mutant. Multiple POU domains have been cocrystallized with DNA and six contact residues identified (Phillips and Luisi 2000). Since the POU domain is highly conserved, these residues were mapped onto BRN2 and the corresponding residues identified (Supplemental Fig. S4A). The Oct1-DNA cocrystal structure indicates two H bonds between asparagine (N) 455 and an adenine in the DNA major groove (Klemm et al. 1994). This residue is conserved in BRN2 (do Vale Coelho et al. 2016) and in silico modeling suggests that an Alanine (A) substitution would disrupt BRN2 DNA binding (Supplemental Fig. S4B). We therefore generated a corresponding N406A BRN2 mutant and compared its DNA-binding activity to wild-type BRN2 by EMSA using bacterially expressed and purified GST-tagged BRN2 wild-type and N406A mutant (Supplemental Fig. S4C). Two known BRN2 DNA targets from the MITF (Goodall et al. 2008) and Kit ligand promoters (Kobi et al. 2010) were used as radiolabeled probes and anti-BRN2 antibody used to verify that the protein bound to the probe was BRN2. The results revealed that in contrast to the wild-type protein, BRN2 N406A was unable to bind either DNA probe. The impaired DNA binding by the BRN2 N406A mutant was reflected in its reduced capacity to repress an MITF promoter luciferase reporter (Supplemental Fig. S4D) that contains a well-characterized BRN2 binding site (Goodall et al. 2008).

Having established that the N406A mutant failed to bind DNA efficiently, we expressed mCherry-tagged BRN2 N406A in U-2 OS cells and performed live cell imaging following LMI (Fig. 3D). Note that mCherry-BRN2 (Fig. 3E) has a reduced signal to background ratio than GFP-BRN2 (Fig. 3C). For the result, it was clear that whereas BRN2 wild type was recruited to sites of DNA damage peaking at 150 sec after damage, BRN2 N406A was recruited at least fourfold less (Fig. 3D,E; Supplemental Movies S3, S4). Significantly, BRN2 recruitment to sites of DNA damage was enhanced using glutamic acid phospho-mimetic substitutions in the two N-terminal p38 phosphorylation sites (Fig. 3F) but was prevented using the S91A,S96A double mutant, consistent with modification of these residues by p38 or other kinases regulating the ability of BRN2 to bind DNA as observed in vitro (Fig. 2G,H).

### BRN2 enhances recruitment of Ku to sites of DNA damage

Given the recruitment of BRN2 to sites of DNA damage induced by LMI or UV as well as the ability of BRN2 to interact directly with Ku70/Ku80, we next assessed whether the expression of BRN2 could impact the recruitment of the Ku complex to damaged DNA. Cells were transfected with a Ku80-GFP expression vector and recruitment to LMI- or UV-induced damage observed over time. The results revealed that prior depletion of BRN2 led to a moderate reduction in Ku80 recruitment to LMI-induced DNA damage (Fig. 3G), a result recapitulated using UV-induced damage (Fig. 3H). In contrast, ectopic expression of BRN2 enhanced Ku80 recruitment to LMI-induced damage, an effect substantially diminished using the BRN2 N406A non-DNA-binding mutant (Fig. 3I). No effect of BRN2 depletion was observed for recruitment of MRE11-GFP or CtIP-GFP (Supplemental Fig. S4E), suggesting that the effect of BRN2 on Ku80 recruitment to damage was specific. Significantly, while recruitment of Ku80-GFP to LMI-induced damage was stimulated by wild-type BRN2 and the S91E,S96E mutant, this effect was abolished using the S91A,S96A mutant that diminished Ku80 recruitment to damage (Fig. 3J, top panel). Note that wild-type BRN2 and the S91,S96 mutants were expressed to similar levels and the level of Ku80-GFP was unaffected by BRN2 (Fig. 3J, bottom panel). Collectively, these data suggest that one role for BRN2 is to facilitate recruitment of Ku to sites of damage.

Although BRN2 could increase recruitment of Ku to sites of DNA damage, in preliminary experiments we found no evidence that BRN2 could affect the efficiency of DDR using a variety of assays including examining the rate of repair of UVB-induced cyclobutane pyrimidine dimers (CPDs) (Supplemental Fig. S4F) or (6-4) pyrimidine-pyrimidone photoproducts (6-4PPs) and repair of DSBs generated at Ase1 or Sce1 cleavage sites in reporter cells. We therefore hypothesized that rather than affecting repair efficiency, BRN2 might impact the quality of repair. Since the Ku70/Ku80 complex is implicated in nonhomologous end-joining (NHEJ) (Lieber et al. 2003), we assessed whether expression of BRN2 would affect the balance of DDR mediated by NHEJ, characterized by 53BP1 foci, versus homologous recombination (HR) marked by RAD51 foci. To this end, we irradiated cells to induce DSBs and determined the numbers of RAD51 or 53BP1 foci per cell over time in control U-2 OS cells or cells expressing BRN2. The results showed that the expression of BRN2 delayed the increase in RAD51 foci induced by γ-irradiation (Fig. 3K) and that by contrast, 53BP1 foci, a hallmark of NHEJ, were significantly increased in cells expressing BRN2 compared with control cells (Fig. 3L). These data are consistent with BRN2 reprogramming repair by promoting a switch away from HR and toward NHEJ by facilitating recruitment of Ku70/80 to DNA damage.

### BRN2 protects melanoma cells from apoptosis following UVB-induced DNA damage

**Figure 4.**
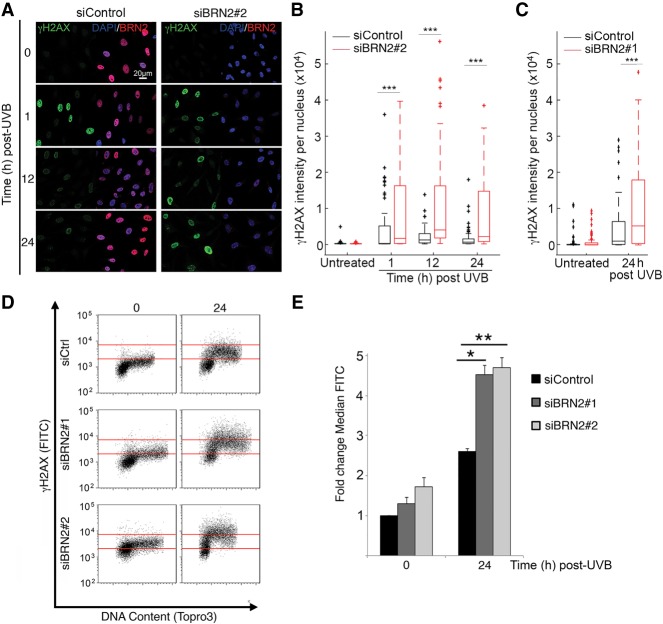
BRN2 depletion causes persistence of γH2AX following UVB irradiation. (*A*) Immunofluorescence of 501mel cells treated with siControl or siBRN2#2 for 48 h prior to UVB irradiation time course as indicated, stained with antibodies against γH2AX and BRN2 with DAPI nuclear counterstain. (*B*) Box plot of quantification of immunofluorescence result shown in *A* using FIJI to analyze the intensity of γH2AX per nucleus. Seventy nine nuclei were analyzed per condition. Asterisks represent *P*-values of unpaired Student's *t*-test between siControl and siBRN2#2 at each time point. (***) *P* < 0.001. (*C*) Quantification of γH2AX intensity per nucleus in cells transfected with siBRN2#1. Seventy-nine nuclei were analyzed per condition. The experiment was performed and analyzed as in *A*. (***) *P* < 0.001. (*D*) Flow cytometry analysis of 501mel cells treated with siControl and two siBRN2 (#1 and #2) for 48 h prior to UVB irradiation. Cells were stained for γH2AX and DNA content 24 h after UVB treatment. The *bottom* red line delineates negative versus positive γH2AX staining, and the *top* red line indicates high positive staining in siControl. (*E*) Quantification of γH2AX-positive cells in *D*. Data represent fold change in percentage γH2AX positive cells compared with untreated siControl: mean ± SD of at least three biological replicates. Intersample comparison by unpaired Student's *t*-test. (*) *P* < 0.05; (**) *P* < 0.01.

UVB principally induces thymine dimers but can also trigger DSBs either directly, through ROS induction, or by conversion of unrepaired dimers. The initial sensing of the DSB by the DDR kinases ATM, ATR, and DNA-PK_CS_ leads to phosphorylation at Ser139 (γH2AX) of the histone protein H2AX that is propagated along the chromatin producing large, extensive foci that can be assayed by immunofluorescence using confocal microscopy or FACS (Huang and Darzynkiewicz 2006; Lukas et al. 2011). However, UVB can also produce a more diffuse γH2AX staining at all phases of the cell cycle that may be generated as a consequence of nucleotide excision repair (Halicka et al. 2005; Marti et al. 2006). We therefore assessed the impact of BRN2 depletion on γH2AX after UVB irradiation. Testing two different siRNAs directed against BRN2 revealed that both reduced BRN2 expression, though siBRN2#2 was more efficient, (Supplemental Fig. S5A). Note that although it has been reported that BRN2 can promote expression of MITF (Wellbrock et al. 2008), we observed no effect of depletion of BRN2 on MITF levels until 72 h post-transfection with siBRN2#2. Since MITF can regulate DDR genes (Strub et al. 2011) and depletion of MITF causes a G1 cell cycle arrest (Carreira et al. 2006), we performed all subsequent experiments at earlier timepoints. We observed no significant effect on the cell cycle 48 h after transfection with siRNAs targeting BRN2 (Supplemental Fig. S5B) and although a recent report suggested that BRN2 can be regulated by E2F1 (Zeng et al. 2018), we saw no significant changes in BRN2 protein level during the cell cycle after Western blotting of extracts from cells synchronized using mitotic shake-off, a technique that avoids the use of cell cycle inhibitors that might stress the cells (Supplemental Fig. S5C). UVB irradiation at a physiologically relevant dose (150 J/m^2^, 1.5-fold the standard erythema dose [SED]; four SEDs are expected to induce moderate erythema on naive white skin, but minimal erythema on previously exposed [tanned] skin) (Diffey et al. 1997) did not affect the levels of BRN2 in cells transfected with a control siRNA or the degree of depletion using siRNAs targeting BRN2 (Supplemental Fig. S5D). We therefore transfected cells with a control siRNA and siRNAs targeting BRN2 and, 48 h later, examined γH2AX induction and resolution after UVB irradiation. Using the more efficient siBRN2#2 revealed that without UVB irradiation BRN2 depletion did not induce a major change in γH2AX levels (Fig. 4A, quantified in B). In contrast, depletion of BRN2 for 48 h led to significantly elevated γH2AX staining at all time points after UVB irradiation. We therefore repeated the analysis using siBRN2#1 that again showed elevated γH2AX levels after UVB irradiation in cells depleted for BRN2, but no significant change in nonirradiated cells (Fig. 4C). A more sensitive flow cytometry assay (Fig. 4D, quantified in E) suggested that depletion of BRN2 could cause a moderate, but not significant, increase in γH2AX staining prior to UVB irradiation but that both BRN2-specific siRNAs led to a significantly increased γH2AX signal compared with the control siRNA 24 h after irradiation.

**Figure 5.**
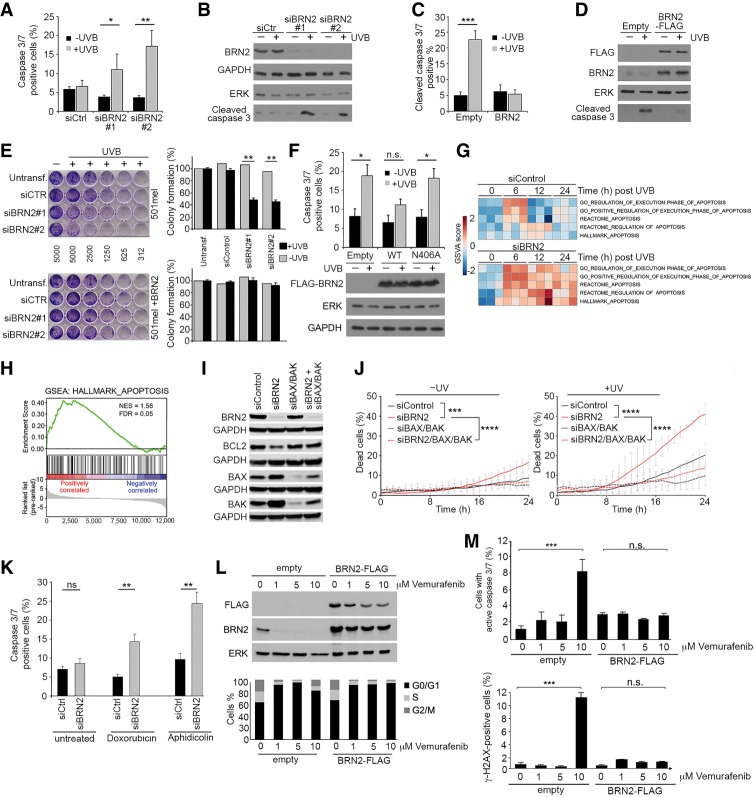
BRN2 protects from apoptosis following UVB- or chemotherapy-induced DNA damage. (*A*) Apoptosis assays using cleaved anti-caspase 3/7 antibody to identify the apoptotic population by flow cytometry 24 h after UVB treatment in 501mel cells transfected with indicated control or BRN2-specific siRNAs. Error bars indicate mean ± S.D. of three biological replicates. Analysis by paired Student's *t*-test. (*) *P* = <0.05; (**) *P* = <0.01. (*B*) Western blot of 501mel cells transfected with control or indicated siRNAs against BRN2 24 h after 150 J/m^2^ UVB irradiation as indicated. (*C*) Apoptosis assays using cleaved anti-caspase 3/7 antibody to identify the apoptotic population by flow cytometry 24 h after UVB treatment in control 501mel cells or cells stably expressing BRN2-Flag. Error bars indicate mean ± SD of three biological replicates. Analysis by paired Student's *t*-test. (***) *P* = <0.001. (*D*) Western blot corresponding to experiment presented in *C* using the indicated antibodies. (*E*) Clonogenic assay using indicated control 501mel cells (*top*) or 501mel cells stably expressing BRN2-Flag (*bottom*) transfected with control siRNA or siBRN2 as indicated. Cells were irradiated with 150 J/m^2^ UVB, immediately plated as indicated, and allowed to grow for 7 d. Numbers between panels indicate numbers of cells plated in each column. Colony formation was quantified and is shown as percentage relative to untreated control. Analysis by paired Student's *t*-test. (**) *P* = <0.005. (*F*) Cleaved caspase 3/7 flow cytometry assay in 501mel cells stably infected with lentivirus producing mCherry-P2A-Flag-BRN2 wild-type, mCherry-P2A-Flag-BRN2 N406A, or mCherry-P2A-Flag-Control vector as indicated. Western blot of cells probed with anti-Flag antibody, anti-ERK or anti-GAPDH is shown *below*. Data present mean ± SD of at least three biological replicates. Analysis by paired Student's *t*-test. (*) *P* = <0.05. (*G*) Heat map showing gene set variance analysis (GSVA) for gene sets related to apoptosis. Data from triplicate RNA sequencing (RNA-seq) of 501mel cells transfected with siControl or siBRN2 for 24 h prior to a time course following 150 J/m^2^ UVB irradiation as indicated. (*H*) Gene set enrichment analysis (GSEA) for HALLMARK_APOPTOSIS gene set plotted by enrichment of gene expression in siBRN2 transfected cells compared with siControl-treated cells after UVB irradiation. Cells were treated with siRNA 48 h prior to UVB irradiation. (*I*) Western blot showing expression of BRN2, BAX, and BAK in 501mel cells 48 h after transfection with siRNAs specific for each gene as indicated. (*J*) IncuCyte quantification of cell death determined by the ratio of SYTOX orange (dead cell count) to SYTO16 green (total cell count)-positive 501mel cells. Forty-eight hours after transfection with siRNAs specific for each gene as indicated, cells were UV-treated (150 J/m^2^), and cell death was determined over time. Mean and SD from three separate experiments are shown. Statistical analysis was performed using Wilcoxon matched-pairs signed rank test with two-tailed *P*-values. (***) *P* < 0.001; (****) *P* < 0.0001. (*K*) Flow cytometry assay showing cleaved caspase 3/7-positive cells in 501mel cells transfected with BRN2-specific siRNA for 24 h prior to treatment with 1 µM doxorubicin or 1.5 µM aphidicolin for 48 h. Analysis by paired Student's *t*-test. (**) *P* < 0.01. (*L*) Western blot of 501mel cells treated with the indicated concentration of vemurafenib for 48 h (*top* panel) and relative cell cycle distribution measured by flow cytometry (*bottom* panel). (*M*) Flow cytometry assay showing cleaved caspase 3/7-positive (*top* panel) and γH2AX-positive (*bottom* panel) 501mel cells treated with the indicated concentration of vemurafenib for 48 h. Error bars indicate mean ± SD of three biological replicates. Analysis by paired Student's *t*-test. (***) *P* = <0.001; (n.s.) nonsignificant.

If damage remains unresolved, cells may initiate an apoptotic response (Halicka et al. 2005). Although γH2AX can mark DNA damage, the persistence of a diffuse γH2AX signal, as observed in the BRN2-depleted cells (Fig. 4A–C), can also indicate increased apoptosis (Halicka et al. 2005). We therefore examined cells for cleaved caspase 3/7, a key marker of apoptosis, using flow cytometry following UVB irradiation in cells depleted of BRN2. The results revealed a significant increase in apoptotic cells 24 h after UVB treatment following knockdown of BRN2 with two different siRNAs, whereas control siRNA transfected cells were resistant to the induction of apoptosis (Fig. 5A). This observation was confirmed by Western blotting (Fig. 5B), where elevated cleaved caspase 3 was detected in BRN2-depleted cells following UVB irradiation but not before. In reciprocal experiments, stable expression of ectopic BRN2 efficiently suppressed the induction of cleaved caspase 3/7 staining following UVB irradiation (Fig. 5C), a result confirmed by Western blotting (Fig. 5D). Note that the difference in levels of apoptosis in the control cells after UVB irradiation in Figure 5, A and B, compared with Figure 5, C and D, reflects the loss of a proportion of apoptotic cells following transfection with the different siRNAs. BRN2 depletion using siBRN2#1 or siBRN2#2 also led to reduced clonogenic survival after UVB irradiation (Fig. 5E, top panel), an effect that was largely reversed in the cells stably expressing ectopic Flag-BRN2 (Fig. 5E, bottom panel).

To examine the impact of the non-DNA-binding mutant on apoptosis, we generated Flag-tagged BRN2 wild-type or N406A mutant-expressing 501mel cell lines using lentiviruses in which mCherry fluorescent protein is separated from the Flag-BRN2 coding sequence by a P2A self-cleaving peptide. This allows stoichiometric expression of the fluorescent protein and BRN2 and avoids any tag-induced localization artifacts. Cell sorting based on mCherry fluorescence was performed prior to analysis of other markers to ensure that infection with the expression vectors led to a similar efficiency of BRN2 protein expression (Fig. 5F). The results of the apoptosis assays showed that while wild-type BRN2 suppressed the increase in cleaved caspase 3/7 induced by UVB irradiation, the non-DNA binding BRN2 N406A mutant failed to rescue cells from apoptosis. Thus, the ability of BRN2 to bind DNA is necessary for protection from apoptosis following UVB-induced DNA damage. This raised the possibility that BRN2 may impose an anti-apoptotic gene expression program that protects cells from UV-induced DNA damage. Indeed, in response to UV radiation BRN2 is known to regulate *GADD45a*, key sensor of genotoxic stress, in a p53-independent fashion (Lefort et al. 2001; Pedeux et al. 2002). We therefore used a triplicate RNA sequencing (RNA-seq) approach to investigate the transcriptional response to UVB irradiation over time of 501mel cells treated with siControl or siBRN2. Examination of the resulting gene expression programs (Supplemental Table 1) using gene set variance analysis (GSVA) showed that in control siRNA transfected cells apoptosis pathways are transiently increased 6 h after UVB irradiation but return to pre-irradiation levels by 12 or 24 h (Fig. 5G). In contrast, BRN2 depletion led to a more persistent up-regulation of the apoptosis–associated gene expression signatures. Gene set enrichment analysis confirmed a robust up-regulation of genes in the HALLMARK_APOPTOSIS gene set when comparing control versus BRN2-depleted cells 12 h following UVB irradiation (Fig. 5H). Examination of each apoptotic signature revealed that a key set of genes associated with apoptosis were robustly up-regulation or down-regulated following UVB irradiation in the BRN2 depleted cells (Supplemental Fig. S6A; Supplemental Table 2), with some representing genes known to be bound or regulated directly by BRN2 or related factors. For example, *BCL2* and *BRCA1* are directly regulated by BRN3a (Budhram-Mahadeo et al. 1999), a closely related POU domain factor, *GADD45a* is also a known direct target of BRN2 (Lefort et al. 2001), as are *APC*, *APPL1*, and *PMAIP1* (Kobi et al. 2010).

To investigate the possibility that BRN2 was regulating the expression of proapoptotic or antiapoptotic BCL2-family members we examined gene expression in the melanoma cell lines in the Cancer Cell Line Encyclopedia (CCLE). Cell lines were ranked by BRN2 expression that was compared with that of 11 BCL2-related proteins. We noted a good positive correlation with expression of the antiapoptotic *BCL2* gene, while a number of proapoptotic genes such as *BID* and *BAD* were negatively correlated with BRN2 (Supplemental Fig. S6B). Western blotting (Fig. 5I) showed depletion of BRN2 reduced BCL2 expression and increased levels of the proapoptotic family members BAX and BAK. These observations suggested that BRN2-depletion sensitizes cells to UVB irradiation by reducing the threshold for activation of the intrinsic cell death pathway (Kalkavan and Green 2018). To confirm this, we combined depletion of BRN2 with siRNA-mediated silencing of the proapoptotic effectors BAX and BAK and monitored the efficiency of knockdown by Western blotting following UVB irradiation (Fig. 5I). Monitoring cells over time indicated that without UVB irradiation, BRN2 depletion led to a moderate increase in cell death that was reversed by depletion of both BAX and BAK (Fig. 5J, left panel), whereas depletion of BAX and BAK alone had no effect. After UVB irradiation (Fig. 5J, right panel), cell death was substantially increased in cells depleted for BRN2, an effect reversed by depletion of BAX and BAK. Quantification of death 24 h after UVB irradiation is shown in Supplemental Figure S6C. Collectively, these results suggest that BRN2 suppresses death via the intrinsic apoptotic pathway.

Although UVB is important in cutaneous melanoma, we also wanted to determine whether BRN2 could protect from the proapoptotic effects of other kinds of DNA-damaging agents. We therefore exposed cells depleted for BRN2 to sublethal doses of doxorubicin (1 µM; a chemotherapeutic agent that causes DSBs) or aphidicolin (1.5 µM; which induces replication stress) and, 24 h later, assayed for cleaved caspase 3/7. The results (Fig. 5K) revealed that depletion of BRN2 effectively increased the proportion of cells undergoing apoptosis irrespective of the nature of the proapoptotic stimulus.

Since the BRAF inhibitor vemurafenib has been widely used to treat melanoma we also asked whether depletion of BRN2 could affect cell death induced by this drug. Consistent with MAPK signaling being required for BRN2 expression (Goodall et al. 2004b), treatment of BRAF^V600E^ mutant 501mel cells with vemurafenib led to loss of BRN2 (Fig. 5L top left panel) and increased the proportion of cells in G1 at 1 and 5 µM, whereas, at 10 µM, vemurafenib the accumulation of cells in G1 was less pronounced (Fig. 5L, bottom left panel). In contrast, cells stably expressing ectopic BRN2 all accumulated efficiently in G1 irrespective of the concentration of vemurafenib used (Fig. 5L, bottom right panel). Remarkably, while 10 µM vemurafenib induced apoptosis, as detected using cleaved caspase 3/7 (Fig. 5M, top panel) and increased γ-H2AX staining (Fig. 5M, bottom panel), both were suppressed by ectopic BRN2 expression. Thus BRN2 can protect against vemurafenib-induced apoptosis.

Significantly, the antiapoptotic effect of BRN2 was not restricted to melanoma. Treatment of the BRN2-expressing SH-SY5Y neuroblastoma cell line with cisplatin, a first line chemotherapeutic agent for this disease, led to apoptosis that was significantly enhanced by depletion of BRN2 (Supplemental Fig. S6D).

### BRN2 expression correlates with a high level of SNVs in melanoma

**Figure 6.**
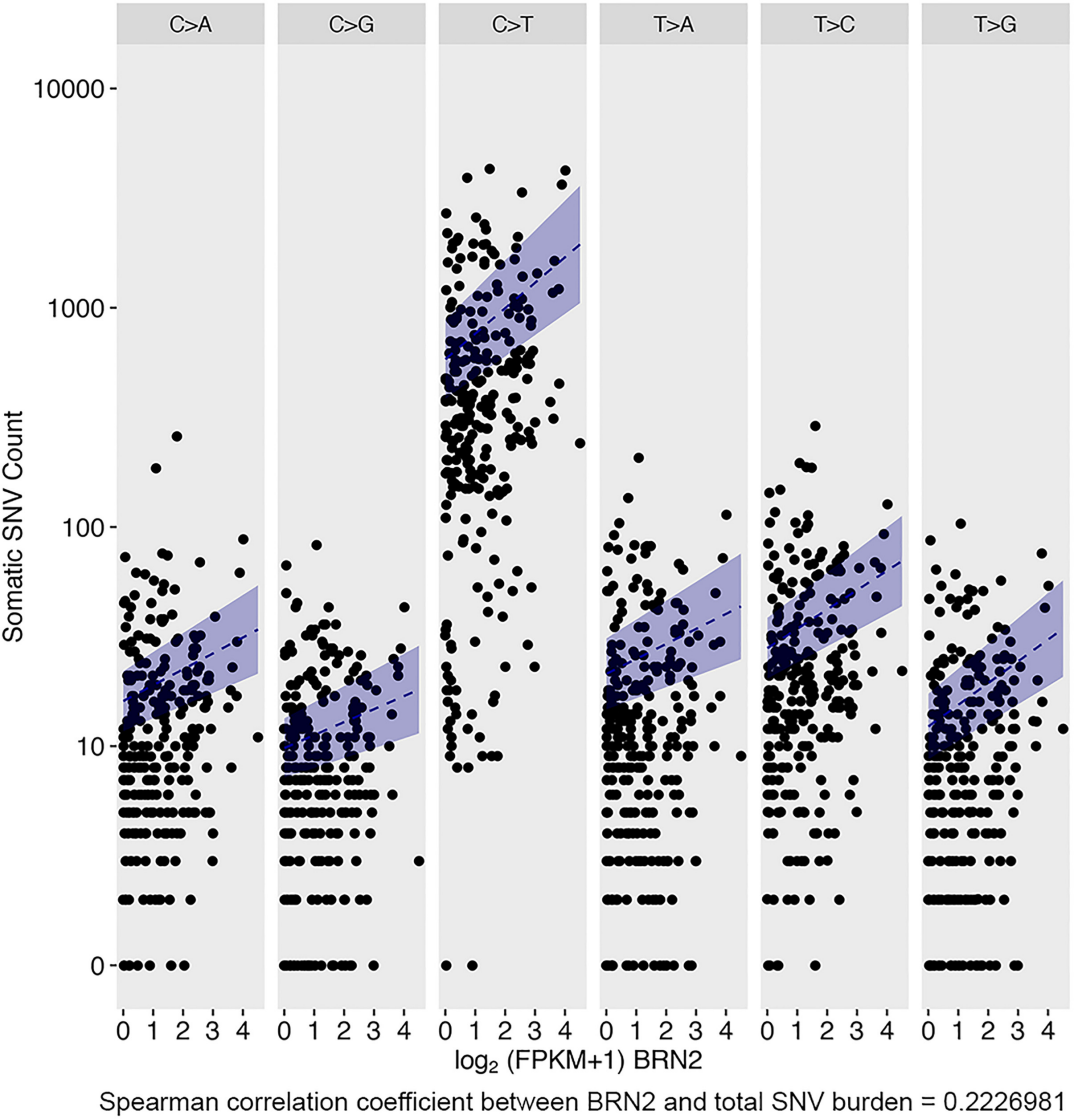
BRN2 expression in melanomas correlates positively with increased mutational burden. SNV counts are plotted against log_2_ (fragments per kilobase per million mapped fragment [FPKM] + 1) BRN2 values. For each SNV class, the blue dashed line (and ribbon) charts the predicted mean mutation burden (and 95% confidence interval) of a patient with the most common constellation of values for clinical variables (see the Materials and Methods) as the log_2_ (FPKM + 1) BRN2 level value increases from the minimum to the maximum observed in the TCGA data set, with all other clinical variables held fixed.

Our results so far suggest that the recruitment of BRN2 to sites of DNA damage and association with Ku may promote NHEJ, an error prone DDR pathway (Lieber et al. 2003), and that BRN2 facilitates survival of cells exposed to DNA damaging agents including UVB and chemotherapy. One anticipated consequence of our observations would be that cells expressing elevated levels of BRN2 might be associated with a high mutational burden. This is particularly relevant in melanoma that exhibits an especially high mutational load that is predominantly caused by solar UV irradiation, with the C > T UV mutational signature increasing during melanoma progression (Hodis et al. 2012; Krauthammer et al. 2012; Alexandrov et al. 2013; Shain and Bastian 2016). To investigate this possibility, we modeled the relationship between somatic single nucleotide variant (SNV) burden and BRN2 expression levels using data from the TCGA melanoma cohort (Fig. 6). As expected, UV and aging-associated C > T transitions dominate the somatic landscape of these tumors. Remarkably, we observed a positive association between BRN2 expression and somatic SNV burden for all six mutation classes (C > A, C > G, C > T, T > A, T > C, and T > G). Negative binomial regression models adjusted for all available clinical variables showed that BRN2 expression level is a statistically significant predictor of SNV burden for all mutation classes (*P* < 0.05 after Benjamini-Hochberg correction for six mutation classes), with the most dramatic correlation being with C > T transition (Supplemental Table 3). We found no correlation between SNV load and the expression of the BRN2-related factors BRN3a (POU4f1) (Supplemental Fig. S7A), BRN3b (POU4f2) which was poorly expressed in most melanomas (Supplemental Fig. S7B), MITF (Supplemental Fig. S7C), or a gene set comprising well-characterized MITF-target genes as a surrogate marker for MITF activity (Supplemental Fig. S7D). Although direct comparisons are difficult, the magnitude of the correlation with somatic SNV burden appears to be similar between BRN2 and that recently reported for melanocortin 1 receptor red hair color variants (Robles-Espinoza et al. 2016) that are important risk factors for melanoma.

## Discussion

**Figure 7.**
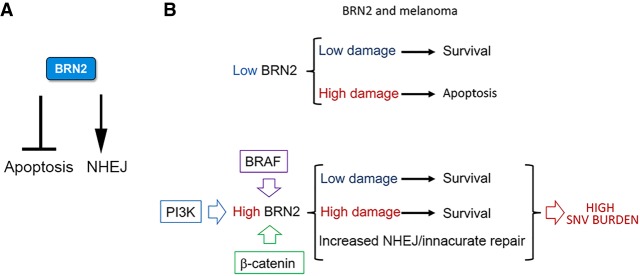
Schematic depicting the role of BRN2. (*A*) BRN2 has two roles: one in suppressing an apoptotic gene expression program, likely indirectly, and a second in promoting NHEJ via its ability to recruit Ku to sites of DNA damage. (*B*) Cells with low levels of BRN2 will be sensitive to apoptosis in response to DNA damage. If BRN2 expression is elevated in response to activation of MAPK, PI3K or β-catenin signaling, heightened resistance to apoptosis combined with the ability of BRN2 to promote error prone repair via NHEJ might explain the correlation between BRN2 expression and high mutation burden in melanoma.

BRN2 is attracting growing attention not only because of its role in neuronal development and in reprogramming but also because of its increasingly recognized role in a range of cancers. In melanoma, BRN2 is expressed in response to oncogenic signaling downstream from BRAF, β-catenin, or PI3K (Goodall et al. 2004a,b; Bonvin et al. 2012) and is especially recognized as playing a critical role in melanoma invasion (Goodall et al. 2008; Arozarena et al. 2011; Fane et al. 2017; Zeng et al. 2018; Thurber et al. 2011). The increased expression of BRN2 that occurs as cells become invasive may reflect BRN2 up-regulation by PI3K signaling that is known to increase as melanoma cells undergo a transition in situ to invasion (Davies 2012; Cho et al. 2015). However, while signaling pathways regulating BRN2 expression have been identified, how BRN2 protein function is regulated has been largely neglected. Here we provide several new insights into BRN2's role and regulation (Fig. 7) that challenge the widely held view that BRN2 acts uniquely as a tissue-restricted transcription regulator.

First and most surprisingly, by AP-MS, we did not detect BRN2 interaction with the repertoire of transcription cofactors such as the SWI/SNF or NuRD complexes or any histone-modifying proteins usually associated with regulators of gene expression. As our AP-MS protocol has been designed for the study of chromatin-associated proteins (Lambert et al. 2014, 2015), the absence of transcription cofactors is unlikely to be due to technical issues. Still, we found BRN2 to interact with CREB and CREM, two bZIP family transcription factors, raising the possibility that BRN2 regulates gene expression by facilitating recruitment of other sequence-specific DNA-binding transcription factors to regulatory elements. Validation of this hypothesis will require comprehensive genome-wide analysis of BRN2 occupancy and biochemical characterization of its capacity to synergize with other transcription regulators in DNA binding and control of BRN2's associated gene expression program. Nevertheless, our observations already suggest that while BRN2 clearly is a sequence-specific regulator of gene expression, it is not a canonical transcription regulator in the cell lines that we used.

Second, we found that BRN2 copurifies with a network of DDR factors implicated in several types of DNA damage. While association with many of these DDR proteins may well be indirect, for example via common association with chromatin, we showed direct interaction with both Ku70/Ku80 and PARP1. Consistent with this observation, BRN2 colocalizes with sites of DNA damage within a few seconds of UVB irradiation and its recruitment is dependent on the activity of PARP1 that plays a key role in chromatin unfolding during damage repair (Strickfaden et al. 2016). Several other transcription factors are recruited to sites of damage in a PARP-dependent fashion (Izhar et al. 2015). However, while it has been proposed that such damage-associated transcription factors may play a role in facilitating the chromatin remodeling necessary for efficient DDR, their role in the DNA damage response has not been established. Our results showing that BRN2 enhances recruitment of Ku70/80 to sites of damage and appears to direct the repair process away from HR towards NHEJ therefore represents a significant advance in our understanding of how transcription factors may play a role in the DNA damage response. This is consistent with in vivo observations, where BRN2 is expressed in melanoma cells with low levels of MITF, a transcription factor associated with proliferation. As such slow-cycling BRN2-expressing cells would be able to undertake NHEJ, but would be less able to use homologous recombination to repair damage since this occurs in G2 after DNA replication. Moreover, unlike some other transcription factors recruited to sites of DNA damage, such as E2F1 or ATF2 (Bhoumik et al. 2005; Chen et al. 2011), BRN2 recruitment requires an intact DNA-binding domain. As such, it is also possible that DNA binding by BRN2 may also modulate the generation of RNA that is increasingly recognized as contributing to efficient DDR (Marnef et al. 2017; Lu et al. 2018).

Third, BRN2 suppresses a proapoptotic gene expression program, with depletion of BRN2 leading to increased apoptosis in response to a range of DNA-damaging agents, including UVB, doxorubicin, and aphidicolin. Importantly, ectopic expression of BRN2 also suppressed apoptosis arising as a consequence of BRAF inhibition by vemurafenib that decreases expression of endogenous BRN2. Since the increased apoptosis observed following depletion of BRN2 could be suppressed by silencing the BCL2 effector proteins BAX and BAK, these results suggest that BRN2 is a generic suppressor of the intrinsic apoptosis pathway irrespective of whether cells are exposed to DNA damaging agents or targeted therapies. The identification of BRN2 as a key antiapoptotic factor in melanoma is consistent with its up-regulation by PI3K signaling (Bonvin et al. 2012) that is known to suppress apoptosis in general (Kennedy et al. 1997) as well as in melanoma (Stahl et al. 2004). This is important, as melanomas and likely other BRN2-expressing cancers exhibit high levels of phenotypic heterogeneity, with BRN2 expression within tumors restricted to a subpopulation of cells associated with invasion (Goodall et al. 2008; Pinner et al. 2009; Zeng et al. 2018). Notably, while BRN2 and MITF are coexpressed in cells in monolayer culture, in vivo the two transcription factors are expressed in a mutually exclusive pattern in cells (Goodall et al. 2008). Since MITF has been implicated in the transcription of *BCL2* (McGill et al. 2002; Thurber et al. 2011) and DDR genes, including *BRCA1* (Beuret et al. 2011; Strub et al. 2011), but has yet to be implicated directly in DNA repair, it suggests that in vivo BRN2 and MITF may play complementary roles in cell survival in response to stress and DNA-damaging agents. Note that while MITF and BRN2 are coexpressed in the cultured melanoma cells used in this study, we observed no effect of depletion of BRN2 on MITF expression at the time points used for the DNA repair or apoptosis assays. However, examining published ChIP-seq (chromatin immunoprecipitation [ChIP] combined with high-throughput sequencing) data sets documenting BRN2 genome-wide binding during neuronal reprogramming did not reveal significant binding of BRN2 to the apoptosis-associated genes identified here as deregulated by BRN2 silencing, with the exception of *BCL2* (Lodato et al. 2013; Wapinski et al. 2013; Mistri et al. 2015; Xue et al. 2016). This may mean that BRN2 largely imposes its antiapoptotic gene expression program indirectly. As such, understanding the molecular mechanisms by which BRN2 can control the apoptotic response will represent an important focus for future research.

Fourth, we show that BRN2 interaction with DNA and with its associated factors is regulated. Deletion of the N-terminal region of BRN2, predicted to be a highly unstructured domain, enhanced BRN2 copurification with a range of DDR factors and increased its ability to bind DNA and, by implication, to regulate transcription. Significantly, mutation of the p38 phosphorylation sites at S91 and S96 to alanine abrogated both the ability of BRN2 to be recruited to sites of DNA damage and its capacity to enhance Ku recruitment to DNA damage, while their substitution with glutamic acid increased DNA binding. However, while p38 signaling is likely to play a key role in facilitating BRN2's role in DDR, the rapid recruitment of BRN2 to damaged DNA and the coimmunoprecipitation of Ku80 with BRN2 in non-UV irradiated cells suggest that additional mechanisms operate to maintain BRN2 in an open conformation, thereby allowing it to access rapidly sites of DNA damage. The identification of signals beyond p38 that may regulate the activity of BRN2 therefore remains a key issue with relevance to both its capacity to repair DNA damage and its ability to regulate transcription and its antiapoptotic function.

Fifth, we show that BRN2, but not MITF, expression correlates with increased SNV burden. Since the mutation burden, including UV signature mutations, increases at all stages as melanoma progresses from benign lesion through to metastasis (Shain et al. 2015), BRN2 expression in melanoma may provide a protective mechanism against apoptosis triggered by DNA damage arising in the primary sun-exposed lesion or in cells undergoing replicative stress as the tumor expands. Such a role for BRN2 in melanoma and other cancers is compatible with the proposed function of BRN2 as a prosurvival factor in the developing nervous system (Schonemann et al. 1995). We view it likely that the increased mutation burden associated with BRN2 expression may in part be a consequence of its prosurvival function: By promoting survival of cells with increased damage, BRN2 may potentiate the expansion of cells with a higher SNV burden. However, by promoting NHEJ, an error-prone DDR mechanism, BRN2, may also contribute to the mutation burden in melanoma. As a consequence, the association between BRN2 and higher levels of genetic diversity within tumors may impact the probability of therapeutic resistance emerging. Moreover, under conditions of nutritional stress, MITF is down-regulated, but BRN2 expression is maintained (Falletta et al. 2017). An increase in mutation load under stress conditions, in part facilitated by BRN2 expression, may provide an evolutionary advantage for cells within the tumor where increased genetic diversity within the population may facilitate survival. Whether expression of BRN2, as an effective biomarker for a high mutation burden, may also be useful in decisions regarding patient stratification for immunotherapy—where some studies have suggested that a high mutation load may be important (Snyder et al. 2014; Van Allen et al. 2015; Lauss et al. 2017; Morrison et al. 2018)—remains to be determined.

Finally, while we focused here on the role of BRN2 in melanoma, we observed that depletion of BRN2 in neuroblastoma cells following cisplatin treatment increased apoptosis, indicating that the ability of BRN2 to modulate the apoptotic response is not restricted to melanoma. As such, our work may have significant implications for a range of cancers where BRN2 is expressed, including small cell lung cancer, glioblastoma, neuroblastoma, and neuroendocrine prostate cancer.

## Materials and methods

### Cell culture and reagents

501mel and stable, inducible BRN2 Flag 501mel melanoma cell lines were cultured in RPMI-1640, U-2 OS osteosarcoma, Ku80-EGFP tagged XR-V15B cells (a gift from Dik van Gent, Erasmus MC, Rotterdam, The Netherlands), and SH-SY5Y neuroblastoma cells were grown in in Dulbecco's modified Eagle medium (DMEM) (Gibco, Thermo Fisher). Cells were cultured as monolayers in 10% CO_2_ at 37°C. All media contained GlutaMAX and Phenol Red and were supplemented with 10% fetal bovine serum and 1% penicillin–streptomycin (10,000 U/mL; Gibco, ThermoFisher) except where indicated. Parental 501mel cells were authenticated by Eurofins-Genomics using a 21-locus single-PCR protocol; all of the cell lines were tested monthly for mycoplasma. The chemotherapy treatments were performed using 1 µM doxorubicin, 1.5 µM aphidicolin, and 1 µg/mL cisplatin for 24 h or with a dose curve of vemurafenib over 48 h, as indicated. For UVB treatment, cells were cultured in individual 35-mm or 60-mm plates. Prior to UVB irradiation, the medium was exchanged for PBS. The plate, with lid removed, was placed in a custom-made chamber and subjected to sham treatment or irradiated with 150 J/m^2^ UVB using a 302 nM bulb (UVM-24 EL series UV 4W; UVP, LLC) calibrated using a light detector at the appropriate wavelength. The medium was then replaced and cells were incubated for remainder of the time course.

### Flow cytometry and apoptosis

Cells at 70%–80% confluency were washed in PBS, trypsinized and resuspended in staining solution SS5 (5% FBS, 2 mM EDTA, 0.01% NaNH_3_ in PBS) and centrifuged. Cells were fixed by resuspending the cell pellet in 70% EtOH in PBS and incubated on ice for 1 h. The fixed cells were then centrifuged and the pellet resuspended in PBS with RNase A 0.1 mg/mL, 0.05% Triton X100, TO-PRO-3 iodide (642/661), FITC anti-H2AX phospho (Ser139) antibody and incubated for 45 min at 37°C. Cells were washed in PBS and resuspended in 250 µL SS5. Ten-thousand to 50,000 cells were measured by FACS ona BD FACSCanto II (BD Biosciences), and data were analyzed using FlowJo software (7.6.1). Apoptosis was analyzed using CellEvent Caspase-3/7 Green flow cytometry assay kit (C10427 Thermofisher); ROS formation was analyzed using CellROX Green flow cytometry assay kit (C10492 Thermofisher) as per manufacturer's instructions on BS LSRFortessa cell analyzer (BD Biosciences). Flow cytometry data was analyzed using FlowJo 10.4 software.

### IncuCyte analysis

Transfection with siRNA was performed 48 h before UV treatment using Lipofectamine RNAiMAX transfection reagents, as per manufacturer's instructions (Invitrogen). ON-TARGETplus siRNA SMART pools of four oligos for human BAX (L-003308-01), human BAK (L-003305-00), and human nontargeting pool (SCR; D-001810-10) were purchased from Dharmacon. Cell death kinetics were assessed and analyzed by the IncuCyte S3 live-cell analysis system (Sartorius). Dead cells and total cell numbers were quantitated using SYTOX orange and SYTO16 green dyes (Essen Bioscience). Percentages of the ratio of SYTOX orange to SYTO16 green counts were calculated to show percentage cell death.

### LMI and UV laser irradiation

For live-cell imaging, U-2 OS cells were cultured in glass-bottomed dishes (Ibidi, catalog no. 81158). GFP-BRN2 or mCherry-BRN2 constructs or empty fluorescent controls were transfected by FuGENE 6 lipofection according to manufacturer's instructions 24 h prior to irradiation. For fixed imaging, 501mel cells were cultured on glass coverslips to 60% confluency. Cells were incubated with Hoescht 33342 for 1 h at 37°C prior to irradiation. For live imaging, DMEM was replaced with FluoroBrite DMEM (Gibco, Thermo Fisher), supplemented with 10% FBS, 1% penicillin–streptomycin, and 4 mM glutamine. For LMI, cells were irradiated using a Mai-Tai multiphoton laser (Spectra Physics) at 750 nm at 5% power (25 mW at the objective) via a plan-apochromat 63×/1.40 oil DIC M27 objective for live or 5 × 5 tile with plan-apochromat 20×/0.8 M27 objective for fixed, with fully open pinhole on LSM710 confocal microscope (Carl Zeiss AG). Live-cell images were obtained every 10 sec from 30-sec preirradiation to 420 sec after irradiation. For UV laser irradiation, cells were irradiated using a Team Photonic UV laser SNV-04P-100 through an iLas^2^ FRAP head (Cairn Research) connected to Nikon TE-2000 microscope with a Nikon Plan Fluor 20×/0.45 objective (Nikon). Laserized pattern and image acquisition were controlled via the software Metamorph (Molecular Devices). Where indicated, cells were incubated with DMSO or PARP inhibitor (10 µM olaparib) for 3 h prior to and during irradiation. Images were analyzed using Fiji and Matlab software. Background to Stripe intensity ratio was calculated for each time point and expressed as percentage change in intensity compared with background.

### Immunofluorescence of DDR proteins following LMI or UV treatment

Following LMI or UV treatment, coverslips were either fixed in 4% PFA and permeabilized in 0.2% PBS-Triton, or soluble proteins were pre-extracted with cytoskeleton (CSK-T) buffer (10 mM PIPES at pH 7.0, 100 mM NaCl, 300 mM sucrose, 3 mM MgCl_2_, 0.5% Triton X-100) and then fixed in 4% PFA. RNase A (0.3 mg/mL) was added to CSK-T for pre-extraction of RNA-bound proteins when staining for Ku80. All coverslips were blocked in 5% bovine serum albumin (BSA) for 20 min at room temperature. Primary antibodies incubated for 20 min at room temperature or overnight at 4°C following pre-extraction. DAPI and secondary antibodies were incubated for 20 min or 1 h (pre-extraction) at room temperature. Coverslips were mounted on glass slides using Mowiol 4-88 mounting medium (12% [w/v] Mowiol 4-88, 30% [w/v] glycerol, 120 mM Tris at pH 8.5). Slides were imaged with LSM 710 confocal microscopes (Carl Zeiss AG). All washes in PBS, all incubations in PBS with 5% BSA.

### Comparison of RAD51 vs. 53BP1 foci

U-2 OS cells were plated on 20-mm^2^ coverslips in six-well plates and transfected with the indicated plasmid or siRNA. After 48 h, plates were exposed to X-rays at a final dose of 2 Gy using a cesium-137 irradiator at the dose rate of 1.87 Gy/min and then put back in the incubator. At the desired time points, immunofluorescence without pre-extraction was performed as described above. More than 400 nuclei were quantified for each condition. Automatized counting of foci was performed with the Fiji software.

### Cloning

Primers were designed using Primer3 (http://bioinfo.ut.ee/primer3-0.4.0/). Inserts were amplified from p3XFlag-CMV-14_BRN2 wild-type and mutant vectors with Accumprime Taq DNA polymerase High-Fidelity kit (Invitrogen). PCR product was purified with Quick PCR purification kit (Qiagen) according to manufacturer's instructions. Amplified, purified inserts were then cloned into a PiggyBac (PB) transposon system vector pPBhCMV1cHApA-MCS (System Biosciences) kindly gifted by K. Murakami (Surani lab) modified to include a puromycin selection cassette (K. Ngeow). QuikChange Lightning site-directed mutagenesis kit (Agilent Technologies) was used to create all BRN2 point mutants, according to manufacturer's instructions. The mCherry sequence was cloned into p3XFlag-CMV-14 BRN2 wild-type plasmid. BRN2 wild type and N406A were cloned into the pGEX-4T-1 vector (GE Healthcare) for bacterial expression.

### Generation of stable cell lines

The FuGENE 6 lipofection system was used to generate polyclonal stable, doxycycline-inducible BRN2 Flag cell lines by cotransfecting PB Brn2-Flag wild-type or truncation mutants and were cotransfected with the PB transposase vector pPyCAG-PBase and the Tet-On System vector with a Neomycin resistance cassette pPB-CAG-rtTA-IRES-Neo. Transfection reagents were prepared in OptiMEM (Gibco, Thermo Fisher) at a FuGENE to DNA plasmid ratio of 3:1. Following transfection, cell lines were subjected to double selection with 3 µg/mL puromycin (Sigma-Aldrich, p8833) and 750 µg/mL Genetecin (Gibco, ThermoFisher, G418) for 48–72 h until the death of all control 501mel cells treated in parallel. Monoclonal cell lines from BRN2-Flag wild type were established by colony picking and were screened by immunofluorescence. Cells were induced with doxycycline as indicated (Sigma-Aldrich, D9891).

### Lentivirus production

A lentiviral vector (pCSII EF1α) containing the sequence encoding BRN2 wild type or N406A and mCherry fluorescent protein separated by a P2A self-cleaving peptide was used to establish stable cell lines in 501mel and U-2 OS cells. Virus was produced by transient transfection into Phoenix cells with Lipofectamine 2000 (Invitrogen) according to the manufacturer's instructions together with vectors encoding Gag/Pol, Rev, and the vesicular stomatitis virus (VSV) envelope. Virus-containing supernatant was harvested at 48- and 72-h post-transfection, passed through a 0.45-µm filter, and target cells were incubated with limiting dilutions of virus from 1:1 to 1:125 for 72 h. Infection efficiency was measured by flow cytometry using a FACSCantoII (BD Biosciences). Target cells with equivalent infection efficiency were expanded and further sorted by FACS according to mCherry expression to achieve a 100% positive cell population with the same levels of the exogenous protein expression.

### Flag affinity purification

The Flag AP-MS protocol was adapted from Lambert et al. (2014) with slight modifications. Stable cells from two 150-mm plates were pelleted, frozen down, and lysed in 1.5 mL of ice-cold low-salt lysis buffer (50 mM HEPES-NaOH at pH 8.0, 100 mM KCl, 2 mM EDTA. 0.1% NP40, 10% glycerol with 1 mM PMSF, 1 mM DTT, Sigma-Aldrich protease inhibitor cocktail [1:500; P8340] added immediately prior to processing). To aid with lysis, the cells were frozen on dry ice, thawed in a 37°C water bath, and then put back on ice. The samples were sonicated at 4°C using three 10-sec bursts with 2-sec pauses at 35% amplitude. One-hundred units of benzonase was then added, and the lysates were incubated for 1 h at 4°C with rotation. The lysates were centrifuged at 20,817*g* for 20 min at 4°C, and the supernatant was added to tubes containing 25 µL of 50% magnetic anti-Flag M2 beads (Sigma-Aldrich, M8823) slurry prewashed in lysis buffer. Flag immunoprecipitation was allowed to proceed at 4°C for 2 h with rotation. Beads were pelleted by centrifugation at 1000 rpm for 1 min and exposed to a magnet, and the unbound lysate was aspirated and kept for analysis. The beads were demagnetized and washed with 1 mL of lysis buffer and magnetized to aspirate off the wash buffer. The beads were then washed with 1 mL of 20 mM Tris–HCl (pH 8.0) containing 2 mM CaCl_2_, and any excess wash buffer was removed by centrifuging the beads and magnetizing and pipetting off the liquid. The now dry magnetic beads were removed from the magnet and resuspended in 7.5 μL of 20 mM Tris–HCl (pH 8.0) containing 750 ng of trypsin (Sigma-Aldrich, T7575), and the mixture was incubated overnight at 37°C with agitation. After the initial incubation, the beads were magnetized, and the supernatant was transferred to a fresh tube. Another 250 ng of trypsin was added to the mixture and further digested for 3–4 h without agitation. The sample was acidified with formic acid to a final concentration of 2%, and the tryptic digests were stored at −40°C until ready for MS analysis.

### Experimental design for MS experiments

For each analysis, two biological replicates of each bait were processed independently. These were analyzed alongside negative controls in each batch of samples processed. Parental 501mel cells expressing no bait (i.e., empty cell lines) were used. These control cell lines were grown in parallel to those expressing baits and treated in the same manner. To minimize carryover issues on the liquid chromatography, extensive washes were performed between each sample and the order of sample acquisition on the mass spectrometer was also reversed for the second biological replicate to avoid systematic bias.

### Preparation of high-performance liquid chromatography (HPLC) columns for MS

A spray tip was formed on fused silica capillary column (0.75 µm ID, 350 µm OD) using a laser puller (program = 4; heat = 280, FIL = 0, VEL = 18, DEL = 200). 10–12 cm of C_18_ reversed-phase material (Reprosil-Pur 120 C_18_-AQ, 3 µm; Dr.Maisch HPLC GmbH, Germany) was packed in the column by pressure bomb (in MeOH). The column was then equilibrated in buffer A prior to sample loading.

### MS acquisition using TripleTOF mass spectrometers

Five microliters of each sample was directly loaded at 400 nL/min onto the equilibrated HPLC column. The peptides were eluted from the column over a 90-min gradient generated by a NanoLC-Ultra 1D plus (Eksigent) nano-pump and analyzed on a TripleTOF 5600 instrument (AB SCIEX). The gradient was delivered at 200 nL/min starting from 2% acetonitrile with 0.1% formic acid to 35% acetonitrile with 0.1% formic acid over 90 min, followed by a 15-min cleanup at 80% acetonitrile with 0.1% formic acid and a 15-min equilibration period back to 2% acetonitrile with 0.1% formic acid for a total of 120 min. To minimize carryover between each sample, the analytical column was washed for 3 h by running an alternating sawtooth gradient from 35% acetonitrile with 0.1% formic acid to 80% acetonitrile with 0.1% formic acid, holding each gradient concentration for 5 min. Analytical column and instrument performance were verified after each sample by loading 30 fmol of BSA tryptic peptide standard (Michrom Bioresources, Inc.) with 60 fmol of α-Casein tryptic digest and running a short 30-min gradient. TOF MS calibration was performed on BSA reference ions before running the next sample in order to adjust for mass drift and verify peak intensity. The instrument method was set to a data-dependent acquisition (DDA) mode that consisted of one 250-msec MS1 TOF survey scan from 400 to 1300 Da followed by 20 100-msec MS2 candidate ion scans from 100 to 2000 Da in high-sensitivity mode. Only ions with a charge of 2+ to 4+, which exceeded a threshold of 200 cps, were selected for MS2, and former precursors were excluded for 10 sec after one occurrence. The collision energy for each window was set independently as defined by CE = 0.06 × m/z + 4, where m/z is the center of each window, with a spread of 15 eV performed linearly across the accumulation time.

### DDA MS analysis

MS data were stored, searched, and analyzed using the ProHits laboratory information management system (LIMS) platform (Liu et al. 2016). Within ProHits, AB SCIEX WIFF files were first converted to an MGF format using WIFF2MGF converter and to an mzML format using ProteoWizard (version 3.0.4468) (Kessner et al. 2008) and the AB SCIEX MS Data Converter (version 1.3 beta). The mzML and mzXML files were then searched using Mascot (version 2.3.02) and Comet (version 2012.02 rev.0). The spectra were searched with the RefSeq database (version 57, January 30, 2013) acquired from NCBI against a total of 72,482 human and adenovirus sequences supplemented with “common contaminants” from the Max Planck Institute (http://141.61.102.106:8080/share.cgi?ssid=0f2gfuB) and the Global Proteome Machine (GPM; http://www.thegpm.org/crap/index.html). The database parameters were set to search for tryptic cleavages, allowing up to two missed cleavage sites per peptide with a mass tolerance of 40 ppm for precursors with charges of 2+ to 4+ and a tolerance of ±0.15 amu for fragment ions. Deamidated asparagine and glutamine and oxidized methionine were allowed as variable modifications. The results from each search engine were analyzed through TPP (the Trans-Proteomic Pipeline version 4.6 OCCUPY revision 3) (Deutsch et al. 2010) via the iProphet pipeline (Shteynberg et al. 2011). SAINTexpress version 3.3 (Teo et al. 2014) was used as a statistical tool to calculate the probability value of each potential protein–protein interaction from background contaminants using default parameters. The four controls samples were kept uncompressed for SAINTexpress analysis. Two unique peptides ions and a minimum iProphet probability of 0.95 were required for protein identification prior to running SAINTexpress. Gene Ontology analysis was conducted using online tools available at http://pantherdb.org (Mi et al. 2013). Cytoscape 3.6 software (Shannon et al. 2003) to produce the interactome scheme.

### MS data visualization and archiving

Dot plots and heat maps were generated using ProHits-viz (prohits-viz.lunenfeld.ca (Knight et al. 2017)). All MS files used in this study were deposited at MassIVE (http://massive.ucsd.edu; MSV000080598; ftp://massive.ucsd.edu/MSV000080598) and to the ProteomeXchange Consortium (http://proteomecentral.proteomexchange.org; PXD006017).

### RNAi

siRNA oligomers targeting BRN2 were purchased from Dharmacon (siBRN2#1; custom siRNA, target sequence: AAGCGCAGAGCCTGGTGCAGG) and Sigma (siBRN2#2; SASI_Hs01-00196791). siPARP1 (human) was purchased from Qiagen (Qiagen Flexitube siRNA Hs_PARP1_5, catalog no. SI02662989). Adequate knockdown efficiency was confirmed for two siRNA by Western blot and immunofluorescence. 501mel cells cultured in antibiotic free media were cultured to 40% confluence and transfected with control siRNA or BRN2 siRNA using Lipofectamine RNAiMAX as per manufacturer's instructions. Cells were then cultured for up to 72 h, during which time they were treated with UVB as detailed below; media was changed for all cells at first treatment time point.

### Luciferase assay

501mel cells were plated in 24-well plates and transfected by FuGENE 6 lipofection as above in triplicate with 200 ng of MITF promoter-luciferase reporter and 200 ng of pCMV BRN2-Flag vector or pCMV Flag empty control per well. Cells were analyzed using a luciferase assay system (Promega).

### Coimmunoprecipitation

Protocol for coimmunoprecipitation sample preparation as described previously (Lambert et al. 2013). Briefly, cell pellets were snap-frozen on dry ice and lysed by freeze–thaw in 250 µL of lysis buffer (50 mM Hepes-NaOH at pH 8.0, 100 mM KCl, 2 mM EDTA, 0.1% NP40, 10% glycerol, 1 mM PMSF, 1 mM DTT, Sigma protease inhibitor cocktail, P8340; 1:500). Samples were sonicated for 30 sec at 4°C. Samples were incubated with 1 µL of benzonase (250 U/µL; Sigma-Aldrich, E1014) for 1 h at 4°C. Samples were centrifuged at 14,000 rpm in a benchtop centrifuge for 20 min at 4°C, 10% of the supernatant was retained as Input, the remainder was incubated for 2 h at 4°C on a rotator with anti-Flag M2 magnetic beads (Sigma-Aldrich product no. M8823), pre-equilibrated in lysis buffer. The supernatant was retained as Unbound fraction. Following five washes in lysis buffer, the beads were resuspended in equal volumes of 2× Laemmli sample buffer, boiled at 95°C, and prepared for Western blot.

### Western blotting

Cells were lysed in Laemmli sample buffer (62.5 mM Tris-HCl at pH 6.8, 2% SDS, 12.5% glycerol, 0.1% Bromophenol Blue, 1% β-mercaptoethanol), sonicated, and then denatured at 95°C. Whole-cell lysates were resolved using SDS–polyacrylamide gel electrophoresis (SDS-PAGE) in 10% 37.5:1 Bis-acrylamide gels. Proteins were transferred to nitrocellulose membrane and blocked in 5% milk at room temperature prior to incubation with primary antibodies overnight at 4°C. Membranes were incubated in secondary HRP-conjugated antibodies for 45 min at room temperature and processed with Amersham ECL (GE Healthcare, RPN2106) prior to film exposure. PBS-Tween 0.1% was used for all washes and incubations were in 5% milk. ERK is used frequently as a loading control owing to its long half-life, between 68 and 53 h (Schwanhäusser et al. 2011). For experiments showing Western blotting of BCL family members, cells were lysed in cell lysis buffer {50 mM Tris-Cl at pH 7.4, 150 mM NaCl, cOmplete protease inhibitors cocktail [Roche], 1% 3-[(3-cholamidopropyl)dimethylammonio]-1-propanesulfonate [CHAPS]}.

### CPD quantification

Following UVB treatment and BRN2 silencing, DNA from 501mel cells was purified from cell pellets with PureLink Genomic DNA kit (Invitrogen). The concentration of purified DNA was measured by spectrophotometry on a NanoDrop 2000 (Thermo Fisher). For immunodetection of CPDs, 100 ng of DNA was diluted into a final volume of 75 µL of 0.4 M NaOH and 10 mM EDTA. Samples were then denatured by boiling for 10 min at 95°C and then neutralized with 2 M cold ammonium acetate (pH 7.0) on ice. A 96-well dot blot apparatus (Jencons, 286-437 DHM-96) was assembled with three layers of 3-mm Whatman blotting paper and nitrocellulose membrane (GE Healthcare Amersham Protran NC; 45-004-000) presoaked in 6× SSC (SSC 20×: 3 M NaCl, 0.3 M Na_3_C_6_H_5_O_7_ at pH 7.0). Wells were rinsed with 1× TE buffer (10 mM Tris-HCl, 1 mM EDTA) under vacuum. Samples were then pipetted into wells and run through under vacuum. Following one rinse under vacuum with 2× SSC, DNA was fixed to the membrane by heating under vacuum for 2 h at 80°C on a gel dryer. The membrane was washed in PBS-Tween 0.1% (PBS-T), blocked in 5% milk in PBS-T for 30 min at room temperature, and then incubated with primary antibody against CPD overnight at 4°C in PBS-T. Following four 15-min washes in PBS-T, the membrane was incubated with secondary HRP-conjugated antibody for 1 h. The membrane was then incubated with Amersham ECL (GE Healthcare, RPN2106) following four 15-min washes in PBS-T and then exposed to radiographic film. The membrane was then rinsed before incubation with SYBR-Gold (1:10,000 in PBS-T; Invitrogen, S11494) for 20 min. The membrane was then imaged in a GelDoc (Bio-Rad) using UV transillumination. Immunoblot and DNA staining were then analyzed using ImageJ software.

### Bacterial protein expression and purification

Purified N-terminal GST-tagged BRN2 wild-type and mutant proteins were produced using a bacterial expression system (pGEX-4T-1 vector (GE Healthcare)). Vectors were transformed by heat shock into BL21 competent *Escherichia coli*. Colonies were expanded at 37°C in Terrific broth (TB) culture medium (1.2% [w/v] tryptone, 2.4% [w/v] yeast extract, 0.4% [v/v] glycerol, 17 mM KH_2_PO_4_, 72 mM K_2_HPO_4_ in water). Protein production was induced by adding 0.5 mM isopropyl-β D-thiogalactoside (IPTG) to cultures with OD_600_ 0.6–0.8 and incubating overnight at room temperature. Proteins were harvested by sonicating bacterial pellets resuspended in Lysis Buffer (300 mM NaCl, 20 mM Tris at pH 8.0, 5 mM DTT, 1× protease inhibitor) and collecting the supernatant following a 20-min centrifugation at 20,000*g*. Proteins were then purified by overnight GST pull-down with glutathione Sepharose 4B beads (GE Healthcare Life Sciences, 17-0756-01) in lysis buffer. Purified proteins were eluted from beads with glutathione.

For the Ku and PARP pull-down assays BRN2 variants were purified from 800 mL of *E. coli* BL21(DE3) RP (Stratagene), grown at 37°C in Luria broth medium supplemented with 100 µg/mL ampicillin and 25 µg/mL chloramphenicol. At OD600 = 0.5, 0.5 mM IPTG was added to the culture and incubated for 18 h at 16°C. The cell pellet was lysed in PBS300 (1× PBS, 150 mM NaCl, 1 mM EDTA, 1 mM DTT, 0.075% Triton X-100, protease inhibitors). The suspension was lysed using a Dounce homogenizer (15 strokes) and sonicated three times for 30 sec. Benzonase (15 U/mL) and 1 mM MgCl_2_ were added, and the lysate was incubated for 1 h at 4°C on a nutator. Insoluble material was removed by centrifugation at 35,000 rpm for 60 min at 4°C. One milliliter of washed glutathione sepharose (GE Healthcare) beads was added to the supernatant and incubated for 1.5 h at 4°C. The beads were washed three times with PBS300 and incubated on a nutator for 45 min at 4°C with 10 mL of HSP solution (PBS300, 5 mM ATP, 15 mM MgCl_2_). The beads were washed twice with PBS500 (1× PBS, 350 mM NaCl, 1 mM EDTA, 1 mM DTT, 0.05% Triton X-100, protease inhibitors) and once with 1× PBS, resuspended in 1× PBS and 0.05% sodium azide solution, and stored at 4°C.

PARP-1 was purified according to standard procedures (Langelier et al. 2011). His-Ku70/Ku80 were purified from baculovirus-infected Sf9 cells. One liter of Sf9 insect cells (1 × 10^6^ cells per milliliter) were infected with baculovirus for 3 d at 27°C. Cells were harvested and centrifuged at 1000 rpm for 10 min, and the cell pellet was resuspended in 40 mL of P5 buffer (pH 7.0; 50 mM Na_2_HPO_4_-NaH_2_PO_4_, 300 mM NaCl, 5 mM imidazole, 0.05% Triton X-100, 10% glycerol) containing protease inhibitors PMSF (1 mM), aprotinin (0.019 UIT/mL), and leupeptin (1 µg/mL). The cell suspension was lysed using a Dounce homogenizer (15 strokes) and sonicated twice for 30 sec (50% output) per 20 mL of lysate. Benzonase (15 U/mL) and 1 mM MgCl_2_ were added, and the lysate was incubated for 1 h at 4°C on a nutator. Insoluble material was removed by centrifugation at 35,000 rpm for 30 min at 4°C. The supernatant was loaded on a 5-mL Talon column (Clonetech), and the proteins were then washed with P30 buffer and eluted with P500 buffer (pH 7.0; 50 mM Na_2_HPO_4_–NaH_2_PO_4_, 300 mM NaCl, 500 mM imidazole, 0.05% Triton X-100, 10% glycerol). The fractions containing the protein were identified by SDS-PAGE, concentrated using an Amicon ultra-15 column (Millipore), dialyzed in storage buffer (pH 8.0; 20 mM Tris-acetate at pH 8.0, 200 mM KAc, 10% glycerol, 1 mM EDTA, 0.5 mM DTT), and stored at −80°C.

### PARP1 and Ku70/Ku80 pull-down assays

GST pull-down assays using 500 ng of GST alone or GST-BRN2 (wild type and ΔN) and His-Ku70/Ku80 or 750 ng of PARP1 were performed in 500 µL of GSTB buffer (20 mM KPO_4_ at pH 7.4, 0.5 mM EDTA, 10% glycerol, 0.5% NP40, 1 mM DTT, 150 mM KCl, 1 mg/mL BSA). The beads coupled to GST or GST-BRN2 (wild-type and ΔN) were preincubated for 20 min in GSTB buffer at room temperature followed by the addition of His-Ku70/Ku80 or PARP-1 for 20 min at room temperature. Complexes were washed four times with GSTB buffer without BSA and eluted with 30 µL of 2× SDS loading buffer. Proteins were visualized by Western blotting using the indicated antibodies.

### EMSA

DNA probes were designed to cover the BRN2-binding sites on two BRN2 target genes, MITF and KitL. Thirty-base-pair complimentary oligos with CTAG overhang (MITF: 5′-CTAGTTTTTACATGCATAACTAATTAGCTTAGGT-3′; KITL: 5′CTAGCGCACCGGAACTAATTAAAGCAAATTTGGA-3′) were hybridized and then labeled with CTP, 10 mCi/mL [α-^32^P] EasyTide Lead (Perkin Elmer) in NEB buffer 2, 0.2 µg/µL BSA, and 2 nM DTT, dATP, dTTP, dGTP, and Klenow for 30 min at room temperature. DNA probes were then purified using a nucleotide removal kit (Qiagen, 28304). Probes were eluted in 100 µL of water and stored at −20°C. EMSA reaction was carried out in a final volume of 20 µL of bandshift buffer (25 mM HEPES at pH 7.4, 150 mM KCl, 10% glycerol, 5 nM DTT, 0.5 µg/µL BSA, 50 ng/µL dIdC). Proteins were run on Coomassie, quantified by ImageJ, and diluted accordingly; 2 µL was added to duplicate reactions and 1 µL of BRN2 antibody was added to one reaction and incubated for 20 min on ice. One microliter of purified probe was then added, and the mixture was incubated for 20 min on ice. Samples were then run on 8% 55:1 Bis:acrylamide gels in 0.5× TBE buffer. Gels were then dried, and films were exposed to the radioactive gel. Six microliters of 2× Laemmli sample buffer was added to 6 µL of the diluted protein and run on a Coomassie to confirm dilution ratios.

### Alignment and three-dimensional protein structure

POU domain protein sequence homology alignment was aligned to mouse BRN2 (top) using T-Coffee (Notredame et al. 2000) and the POU domains then selected and formatted using the BOXSHADE server (available at https://embnet.vital-it.ch/software/BOX_form.html). To create the Oct1 crystal structure images, the Protein Data Bank (PDB) ID 1Oct (http://dx.doi.org/10.2210/pdb1oct/pdb; Klemm et al. 1994) was downloaded from the PDB (Berman et al. 2000), and images were created using the University of California at San Francisco Chimera 1.11.2 package (Pettersen et al. 2004). Amino acids were numbered according to mouse BRN2 sequence and the mouse OCT1 variant NCBI reference NP_035267.2.

### RNA-seq and bioinformatics

RNA was extracted using the RNeasy kit (Qiagen, 74106) and QC on a Bioanalyzer (for RIN ≥9.5). ERCC ExFold RNA spike-in mixes (Ambion) were added prior to library preparation using the QuantSeq Forward kit (Lexogen, 015.96) using 500 ng of starting material to minimize the PCR amplification step. Samples prepared as biological triplicates were sequenced on HiSeq 2500 (Illumina) carried out using Wellcome Trust Genomic Service, Oxford. The output raw fastq files were trimmed of poly-A using cutadapt (Martin 2011) and mapped using STAR (Dobin et al. 2013) against hg38 (GRCh38, 2015). Counts per gene from STAR were used as input for differential gene expression analysis using EdgeR (Robinson et al. 2010). Reads for each sample set were first filtered for genes whose expression is less than one count per million prior to glmQLFTest. Genes with a *P*-value of ≤0.05 and meet the specific fold-changes were taken for further analysis. Heat maps of RNA-seq samples were generated from the edgeR-library normalized reads of genes whose differential gene expression has a *P*-value of ≤0.05 before further filtering for genes with read counts two or more counts in all the replicates of either treated or control samples. Raw reads were log_2_ transformed, centered normalized around mean and hierarchical clustering performed using complete linkage using Gene Cluster 3.0 (de Hoon et al. 2004). The output matrix was used to generate the heat-map for visualization using TreeView (Saldanha 2004). Gene set enrichment analyses (GSEAs) were carried out using javaGSEA2-3.0 (Subramanian et al. 2005). One-thousand permutations were carried out for each probe gene set. GSVA analyses were performed using the Bioconductor package GSVA (Hänzelman et al. 2013). The gene sets used were obtained from the Molecular Signatures Database (Subramanian et al. 2005). The GSVA matrix was then clustered and displayed as a heat map using Pheatmap (https://cran.r-project.org/web/packages/pheatmap/index.html).

### TCGA bioinformatics analysis

Files with FPKM (fragments per kilobase per million mapped fragment) gene expression measurements were downloaded on November 20, 2017 from TCGA (https://portal.gdc.cancer.gov/projects/TCGA-SKCM) with the following filters: project ID: TCGA-SKCM; sample type: metastatic, primary tumor, additional metastatic; workflow type: HTSeq-FPKM; data category: transcriptome profiling. We added log_2_ (FPKM + 1) values for genes POU3F2 (BRN2, ENSG00000184486), POU4F1 (BRN3a, ENSG00000152192), and POU4F2 (BRN3b, ENSG00000151615) as variables to the negative binomial model previously published (Robles-Espinoza et al. 2016). This left 271 samples in the model, as two did not have available FPKM measurements (TCGA-EE-A3AE and TCGA-GN-A269). We also added log_2_ (mean FPKM [MITF target genes]) as a variable, where MITF target genes = MITF (ENSG00000187098), BCL2 (ENSG00000171791), CDK2 (ENSG00000123374), CDK4 (ENSG00000135446), DCT (ENSG00000080166), HIF1A (ENSG00000100644), MLANA (ENSG00000120215), PMEL (ENSG00000185664), PPARGC1A (ENSG00000109819), PTEN (ENSG00000171862), RAB27A (ENSG00000069974), SHC4 (ENSG00000185634), TRPM1 (ENSG00000134160), TYR (ENSG00000077498), TBX2 (ENSG00000121068), MCOLN1 (ENSG00000090674), DIAPH1 (ENSG00000131504), and MET (ENSG00000105976). For each of the six basic SNV classes (C > A, C > G, C > T, T > A, T > C and T > G) we modeled the relationship between expected somatic SNV burden and these variables using negative binomial regression with a log link, controlling for all available clinical variables in the TCGA cohort, as described previously (Robles-Espinoza et al. 2016). The most common constellation of clinical variables, for which the prediction depicted with the blue dashed line in Figure 6 was performed, consisted of a male patient from the University of Sydney with a nonulcerated tumor in the extremities and an age of diagnosis of 57 yr old, Breslow thickness of 2.5 mm, Clark level IV, and metastasis to a regional lymph node. Log_2_ (FPKM + 1) values for BRN3a and BRN3b were 0.09167 and 0, respectively, and log_2_ of the median of FPKM values of MITF targets was 7.032.

GSEAs were performed using javaGSEA version 2.2.2 software (http://software.broadinstitute.org/gsea/downloads.jsp) with 10,000 permutations and default parameters using the Verfaillie invasive and proliferative gene expression signatures (Verfaillie et al. 2015).

### Data analysis

Visualization of data and statistical analysis other than that generated by AP-MS or RNA-seq was performed using Prism7 software (GraphPad Software, Inc.). FACS data analysis was performed by ordinary two-way ANOVA and Sidak's multiple comparisons test.

### Data

RNA-seq data have been deposited in the Gene Expression Omnibus (GEO) with accession number GSE124761. MS data are available at MassIVE (http://massive.ucsd.edu; MSV000080598; ftp://massive.ucsd.edu/MSV000080598) and the ProteomeXchange Consortium (http://proteomecentral.proteomexchange.org; PXD006017).

## Supplementary Material

Supplemental Material
