# Supplementary material for: BRN2 suppresses apoptosis, reprograms DNA damage repair, and is associated with a high somatic mutation burden in melanoma
Source: Genes Dev. 2019 Mar 1;33(5-6):310–32. doi: 10.1101/gad.314633.118 (PMC6411009; doi:10.1101/gad.314633.118)
Supplement: Supplemental Material [file supp_33_5-6_310__index.html]

BRN2 suppresses apoptosis, reprograms DNA damage repair, and is associated with a high somatic mutation burden in melanoma — Supplemental Material 

# BRN2 suppresses apoptosis, reprograms DNA damage repair, and is associated with a high somatic mutation burden in melanoma

## Supplemental Material

- Supplemental\_Material.docx
- Supplemental\_Data.pdf
- SUPPLEMENTAL\_TABLE3.xlsx
- Supplemental\_Movie\_1.mov
- Supplemental\_Movie\_4.mov
- Supplemental\_Movie\_2.mov
- SUPPLEMENTAL\_TABLE1.xlsx
- Supplemental\_Movie\_3.mov
- SUPPLEMENTAL\_TABLE2.xlsx
