# Supplementary material for: BRN2 suppresses apoptosis, reprograms DNA damage repair, and is associated with a high somatic mutation burden in melanoma
Source: Genes Dev. 2019 Mar 1;33(5-6):310–32. doi: 10.1101/gad.314633.118 (PMC6411009; doi:10.1101/gad.314633.118)
Supplement: Supplemental Material [file supp_gad.314633.118_Supplemental_Material.pdf]

SUPPLEMENTAL MATERIALS TABLE OF CONTENTS

Supplemental Materials and Methods

Supplemental Figures and Movie List

Supplemental Table legends

SUPPLEMENTAL MATERIALS AND METHODS

Primary antibodies used

Protein target	Species	Isotype	Supplier	Product #	Dilution	
					WB	IF
BRN2	rabbit	IgG	Cell Signaling	12137	1:2,000	1:200
ERK2	mouse	IgG2bκ	Santa Cruz Biotechnologies	sc-1647	1:5,000	
ERK2	rabbit	polyclonal	Santa Cruz Biotechnologies	sc-154	1:10,000	
BCL2	mouse	monoclonal	Cell Signaling	15071	1:1000	
BAX	rabbit	polyclonal	Santa Cruz Biotechnologies	sc-493	1:1000	
BAK	rabbit	polyclonal	Santa Cruz Biotechnologies	sc-832	1:1000	
RAD51	rabbit	polyclonal	Santa Cruz Biotechnologies	sc-8349		1:200
53BP1	rabbit	polyclonal	Cell Signaling	4937		1:200
His	rabbit	polyclonal	Clontech	631212	1:2500	
PARP-1 (F1-23)	mouse	monoclonal	Homemade		1:2500	
FLAG® M2	mouse	IgG1	Sigma-Aldrich	F1804	1:10,000	1:500
GAPDH	rabbit	polyclonal	Sigma-Aldrich	G9545	1:5,000	

Ku80	rabbit	IgG	Abcam	ab79391	1:1,000	1:200
Cleaved caspase 3	rabbit	polyclonal	Cell Signaling	9664	1:1000	
CPD	mouse	IgG2a,κ	Cosmo-Bio	CAC-NMDND-001	1:2,000	
Phospho H2AX (S139) (γH2AX)	mouse	IgG1	Millipore	05-636	1:500	1:200
Phospho H2AX (S139) (γH2AX)	rabbit	IgG	Cell Signaling	9718	1:500	1:200
Phospho H2AX (S139) (γH2AX)-FITC	mouse	IgG1	BioLegend	613403	FACS: 1:250	

Secondary antibodies used

**Species**	**Target species**	**Isotype**	**Conjugate**	**Supplier**	**Product #**	**Use (dilution)**
goat	mouse	IgG (H + L)	HRP	Biorad	170-6516	WB (1:10,000)
goat	rabbit	IgG (H + L)	HRP	Biorad	170-6515	WB (1:10,000)
donkey	mouse	IgG (H + L)	Alexa Fluor™ 488	Life technologies	A-21202	IF (1:500)

donkey	rabbit	IgG (H + L)	Alexa Fluor™ 488	Life technologies	A-21206	IF (1:500)
donkey	mouse	IgG (H + L)	Alexa Fluor™ 546	Life technologies	A10036	IF (1:500)
donkey	rabbit	IgG (H + L)	Alexa Fluor™ 546	Life technologies	A10040	IF (1:500)

SUPPLEMENTAL FIGURES

Figure S1

Figure S2

Figure S3

Figure S4

Figure S5

Figure S6

Figure S7

SUPPLEMENTAL MOVIES

(Movie S1) GFP-BRN2^{WT} + DMSO live LMI (GFP_BRN2_WT_DMSO.mov)

(Movie S2) GFP-BRN2^{WT} + PARPi live LMI (GFP_BRN2_WT_PARPi.mov)

(Movie S3) mCherry-BRN2 ^{WT} live LMI (mCherry_BRN2_WT.mov)

(Movie S4) mCherry-BRN2^{N406A} live LMI (mCherry_BRN2_N406A.mov)

Supplemental TABLE 1 Gene expression changes over time following UVB irradiation in cells depleted for BRN2 or cells transfected with control siRNA.

Supplemental TABLE 2. Table 2 shows up-or down-regulated genes implicated in apoptosis after cells were depleted for BRN2 using siRNA and then UVB irradiated. Comparisons are made versus siControl 6 h or 12 h post-UVB irradiation.

Supplemental TABLE 3. Table 3 shows the output from negative binomial regressions with TCGA data, with separate models fitted for each of the six single nucleotide variant (SNV) classes (outcome is SNV count). The exponential of the estimated coefficient, the incident rate ratio, can be interpreted as a multiplicative factor affecting the expected SNV count outcome variable. The accompanying confidence intervals are at 95%.
