# Supplementary material for: BRN2 suppresses apoptosis, reprograms DNA damage repair, and is associated with a high somatic mutation burden in melanoma
Source: Genes Dev. 2019 Mar 1;33(5-6):310–32. doi: 10.1101/gad.314633.118 (PMC6411009; doi:10.1101/gad.314633.118)
Supplement: Supplemental Material [file supp_gad.314633.118_Supplemental_Data.pdf]

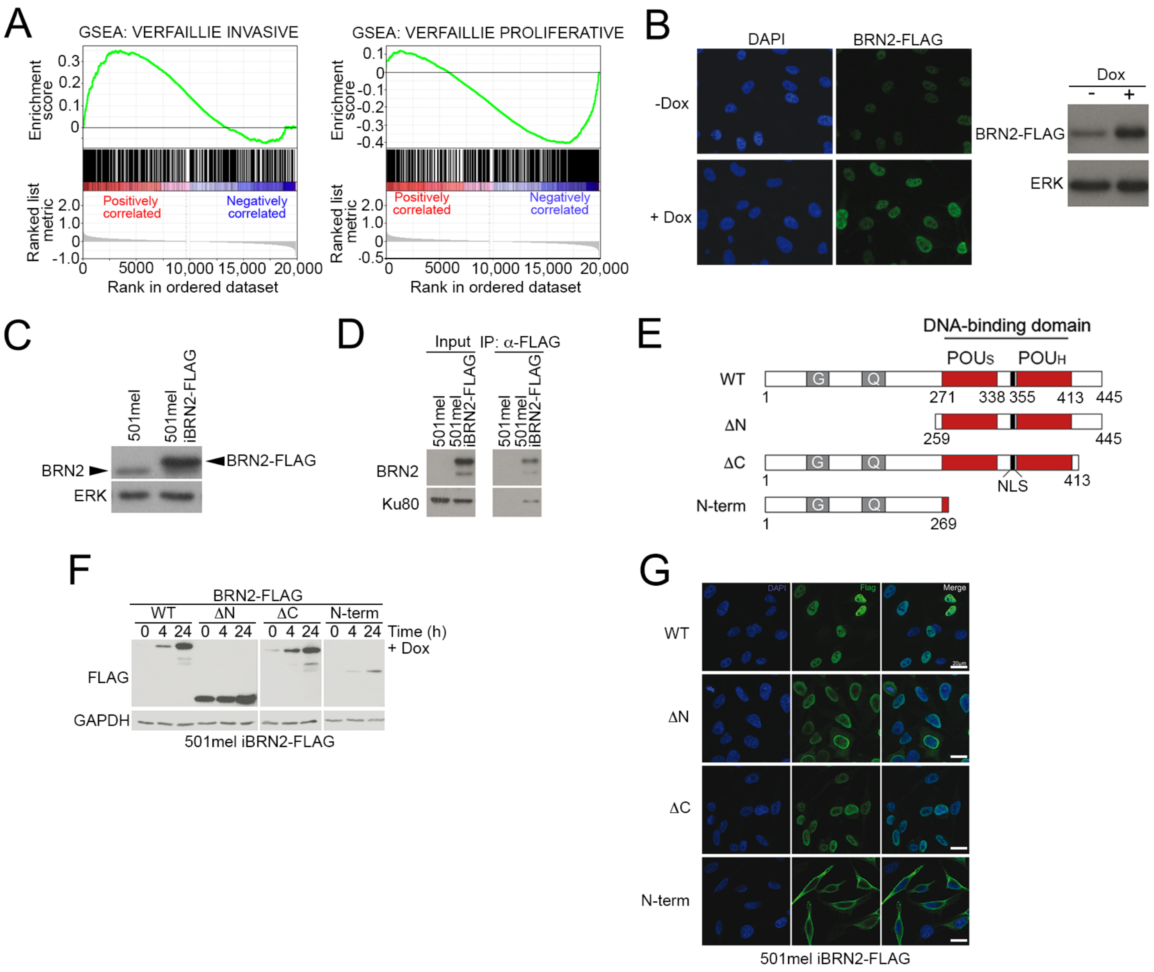

Figure S1. (*A*) GSEA analysis of RNA expression data from the TCGA melanoma cohort showing the enrichments for the Verfaillie Invasive or Proliferative gene expression signatures when comparing the top and bottom 100 melanomas ranked by expression of BRN2. (*B*) Immunofluorescence images of stable PiggyBac BRN2 WT-FLAG (iBRN2) cell line before and after 24 h induction with 10 ng/ml doxycycline together with a corresponding Western blot using anti-FLAG or ERK antibodies of the PiggyBac BRN2 WT-FLAG cell line with and without doxycycline induction. (*C*) Western blot using anti-BRN2 showing expression of endogenous BRN2 versus FLAG-tagged BRN2 in the stable iBRN2 expressing cell line without doxycycline. (*D*) Western blot using indicated antibodies after co-immunoprecipitation of KU80 with BRN2 following immunoprecipitation with anti-FLAG antibody from 501mel or 501mel iBRN2-FLAG without doxycycline as indicated (*E*) Schematic representing full length BRN2 (POU3F2) and truncation mutants. G = polyglycine tract; Q = polyglutamine tract; POU_S = POU specific domain; POU_H = POU homeodomain. Numbers indicate amino acid positions. (*F*) Western blot using indicated antibodies showing expression levels and differential size of BRN2 WT and truncation mutants stably expressed in 501mel cells following induction with 10 ng/ml doxycycline over time. All cell lines were created using the PiggyBac vector as for the cell line shown in (*B*). (*G*) Immunofluorescence of cell lines described in (*F*) after 24 h induction with 10 ng/ml doxycycline. Cells stained with antibody against FLAG, with DAPI nuclear counterstain.

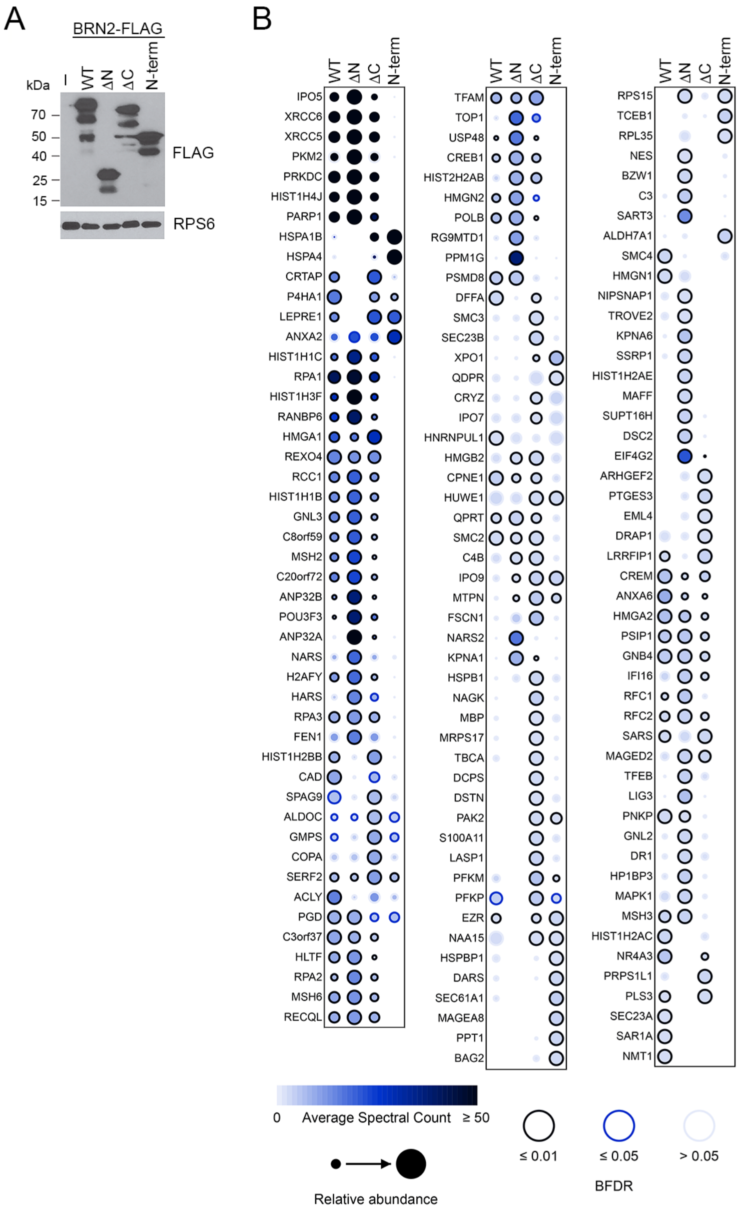

Figure S2. (*A*) Western blot showing relative expression levels of the different FLAG-tagged BRN2 proteins expressed in 501mel cells and immunopurified using anti-FLAG antibody prior to MS analysis. (*B*) Dot-plot of AP-MS data showing results of all BRN2 constructs. Size of dots represents relative abundance, colour of dots represents average spectral count, grey intensity of circle encompassing dot represents the Bayesian False Discovery Rate (BFDR) cut off.

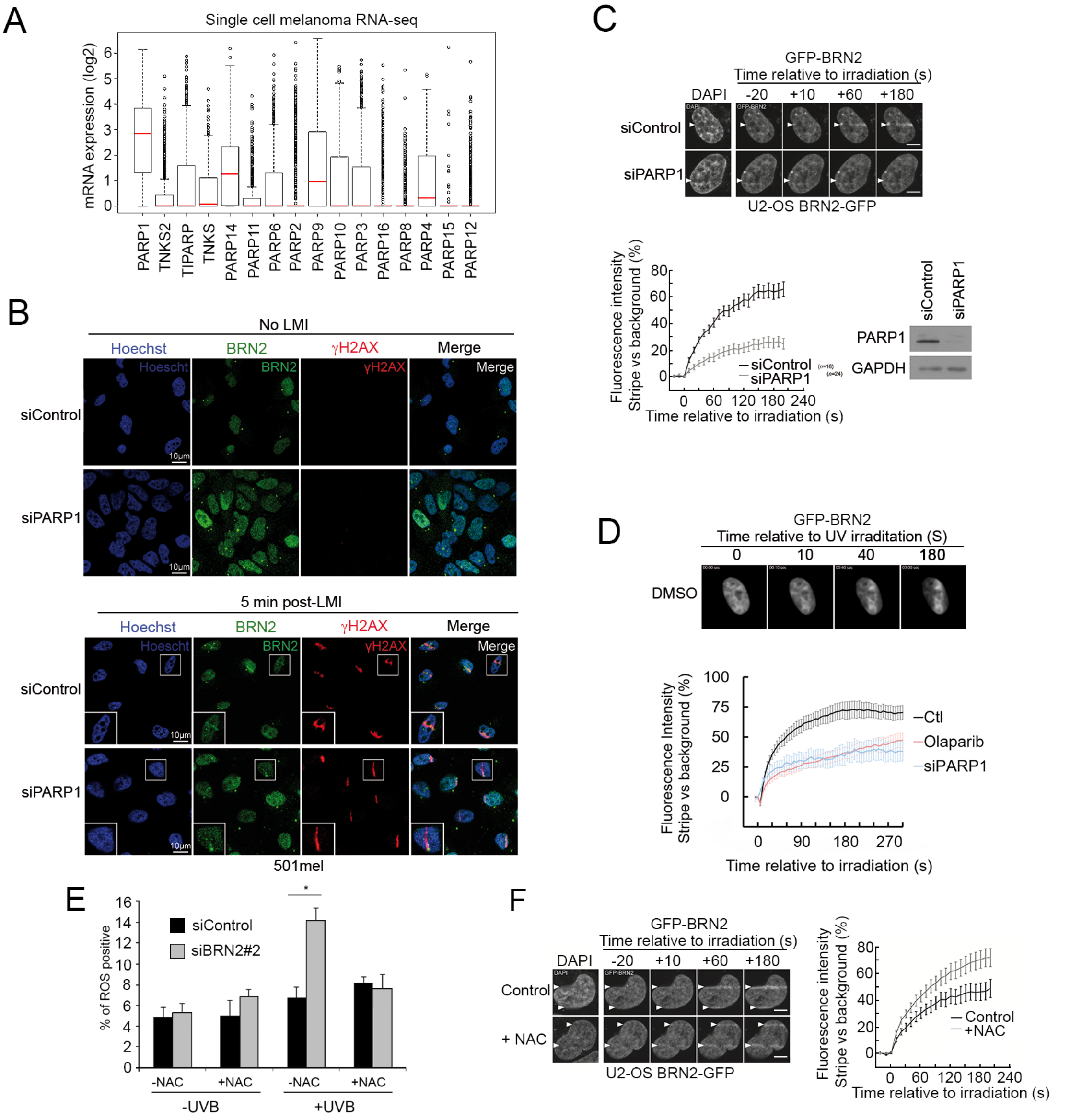

Figure S3. (*A*) Relative expression levels of PARP family members in melanoma. Boxplots represent log2 values of PARP family mRNAs derived from the melanoma single cells RNA-seq dataset described by Tirosh et al (2016). Boxplots represent medians (red lines) with interquartile ranges. Data points that lie outside the whiskers are considered as outliers. (*B*) Immunofluorescence of 501mel before (upper panels) or 5 min after (lower panels) laser micro-irradiation (LMI) treatment. Cells were stained with antibodies against γH2AX and endogenous BRN2 and were either transfected with control siRNA or siRNA-specific for PARP1 48 h prior to LMI as indicted. (*C*) Upper panels show still images from live cell imaging after LMI of U-2 OS cells expressing GFP-BRN2 in cells transfected with control siRNA or siPARP1. Below, timecourse quantification (left) and Western blot (right) indicating efficiency of PARP1 depletion. Error bars indicate +/- SEM. (*N*: Control = 15; Olaparib = 18; siPARP1 = 15). (*D*) UV-microirradiation of U-2 OS cells transfected with GFP-BRN2. Upper panel represents still images at indicated timepoints, with real time quantification shown below including cells treated with PARPi (Olaparib 10 mM) or transfected with siPARP1 48 h prior to UV irradiation. Error bars indicate +/- SEM. (*E*) Quantification of ROS by flow cytometry 24 h after UVB treatment with or without treatment with 6 mM NAC using control 501mel cells or cells transfected with siBRN2. Error bars indicate mean +/- S.D. of three biological replicates. Analysis by paired Student's t-test. * *P* = <0.039. (*F*) Snapshots taken at indicated times of real time imaging of LMI of U-2 OS cells transfected with BRN2-GFP treated with 6 mM NAC as indicated with real time quantification shown to the right. Error bars indicate +/- SEM. (*N*: Control = 14; NAC = 19).

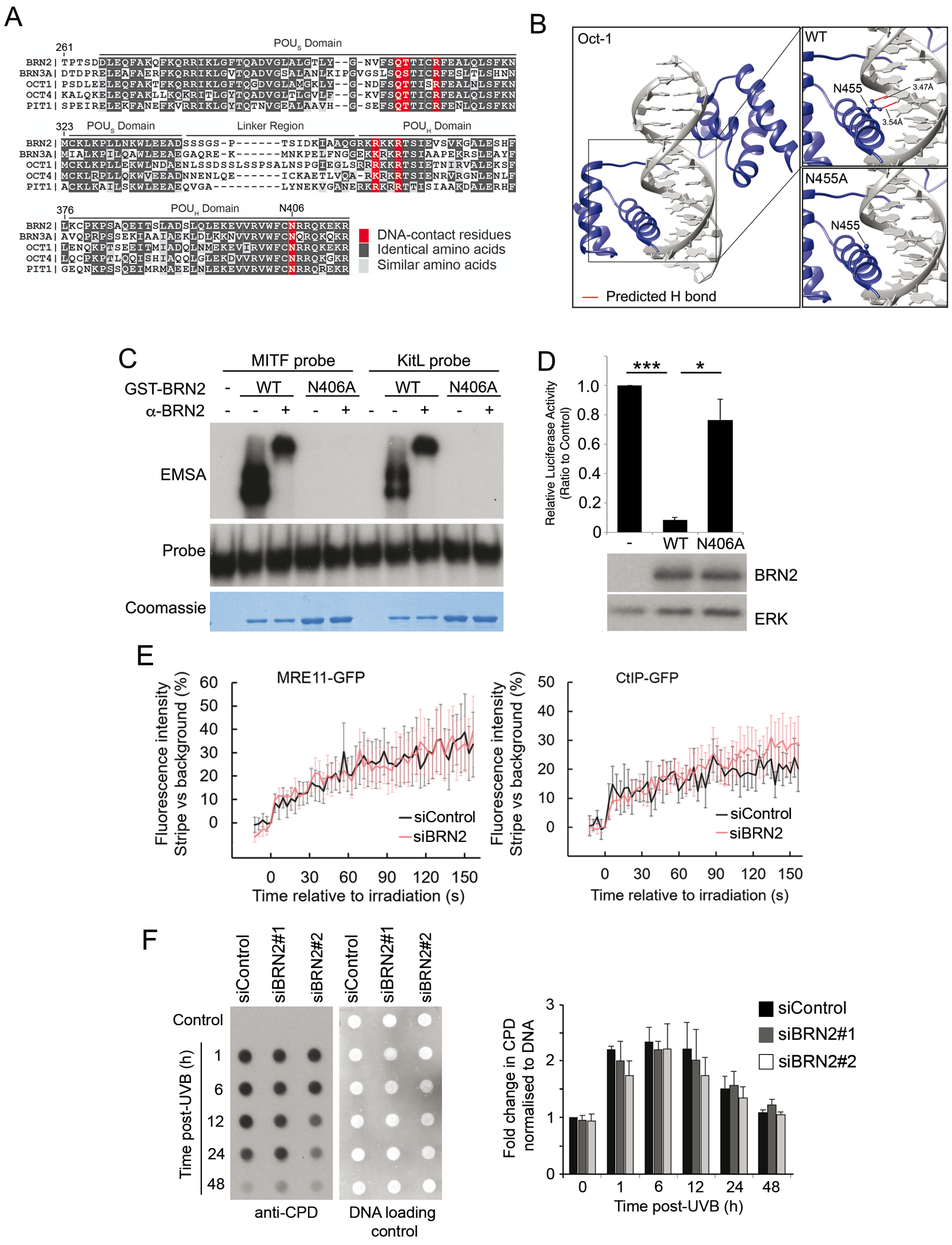

Figure S4. (*A*) Schematic showing POU domain sequence homology between mouse BRN2, BRN3A, OCT1, OCT4, PIT1. Red = DNA-contact residues; Dark Grey = Identical amino acids; Light Grey = similar amino acids. (*B*) Images showing co-crystal structure of Oct-1 with DNA. Upper inset: Residue N455 on Oct1 based on NCBI reference sequence NP_035267.2 (corresponding to N406 in BRN2) makes two predicted H bonds (red lines) with the DNA. Lower inset: Residue N455 mutated to Alanine (N455A) demonstrating predicted loss of H bonds. (*C*) Electrophoretic mobility shift assay (EMSA) of purified GST-BRN2 WT or GST-BRN2 N406A incubated for 30 min with radiolabelled DNA probes corresponding to validated BRN2 binding motifs. Upper panel shows bound probe. Addition of anti-BRN2 antibody shows super-shift demonstrating specificity. Coomassie staining of SDS-Page gel run in parallel to show protein used for EMSAs. (*D*) Activity of an MITF promoter-luciferase reporter in 501mel cells co-transfected with an Empty-FLAG or BRN2 WT-FLAG or BRN2 N406A-FLAG expression vectors. Results are expressed as mean ± SD of three biological replicates. Inter-sample comparison by unpaired Student's t-test (* *p<0.05; *** p<0.001*). Western blot shows BRN2-FLAG expression levels. (*E*) Time course of MRE11-GFP or CtIP-GFP co-localization with LMI-induced DMA damage over time in 501mel cells transfected with control siRNA or siBRN2. Error bars indicated +/- SEM. (*N*: MRE11-GFP siNT = 5; MRE11-GFP siBRN2 = 9; CtIP-GFP siNT = 5; CtIP-GFP siBRN2 =5). (*F*) CPD repair efficiency determined using anti-CPD antibody to probe a dot blot of DNA isolated at indicated times from 501mel cells irradiated with 150 J/m^2 UVB as Indicated. Cells were transfected with siControl or siBRN2 as indicated 48 h prior to irradiation. Quantification is shown to the right. N=4. Error bars indicate +/- S.D. BRN2–depletion did not significantly alter the CPD repair rate as determined using a paired Student's t test.

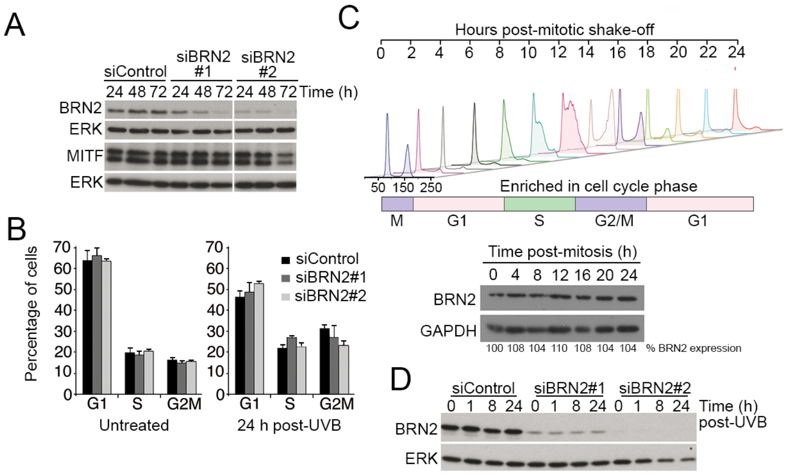

Figure S5. (*A*) Representative Western blot using indicated antibodies showing validation of siBRN2#1 and #2 in 501mel cells compared siControl treated cells. Time corresponds to hours post transfection of siRNA. (*B*) Flow cytometry analysis of cell cycle distribution of 501mel cells transfected with control or indicated BRN2 siRNAs for 48 h. Data presented are mean ± SD of three biological replicates. (*C*) Mitotic shake-off experiment to show expression of BRN2 in the cell cycle. Poorly attached cells in mitosis were removed from the plastic by mild shaking and re-plated (Time 0). Their DNA content was determined at different times after plating by flow cytometry (upper panel), with the corresponding Western blot at selected time points shown below. (*D*) Western blot of 501mel cells transfected with control siRNA or indicated siBRN2 for 48 h prior to UVB irradiation (150 J/m^2) and harvested over time.

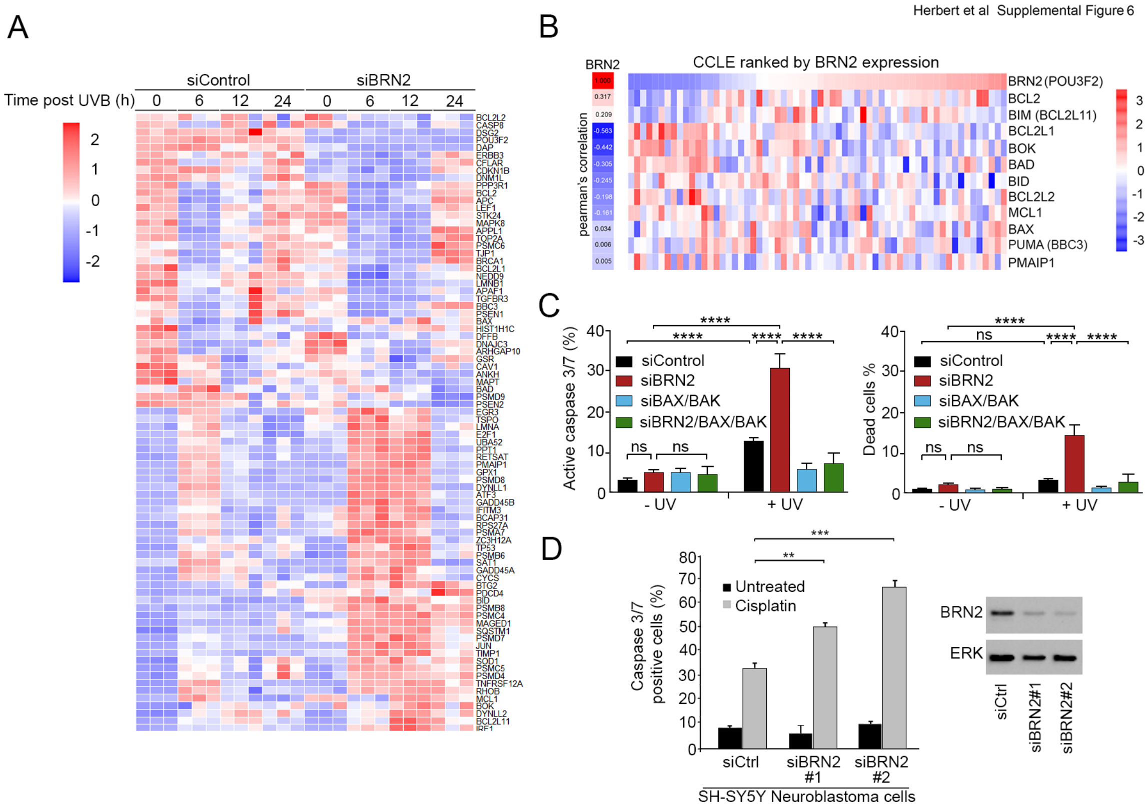

Figure S6. (*A*) Heatmap of mRNA levels corresponding to indicated apoptosis-associated genes derived from triplicate 3′ RNA-seq of cells transfected with siRNA control or siBRN2 for 24 h followed by UVB irradiation. Cells were harvested at indicated times after UVB treatment. (*B*) Relative expression of indicated genes in the CCLE melanoma cell lines ranked by *BRN2* expression. Spearman's correlation between expression of indicated genes and BRN2 is shown in the left column. (*C*) Flow cytometry assay showing cleaved caspase 3/7-positive (left panel) and dead cells (right panel) 24 h after UV irradiation (150 J/m^2) of 501mel cells. Where indicated, cells were pre-treated with indicated siRNA for 48 h. Data are represented as mean ± SD of three independent experiments and analyzed using ordinary two-way ANOVA and Sidak's multiple comparisons test. ns, non-significant. **** *P* = <0.0001. (*D*) Flow cytometry assay showing cleaved caspase 3/7-positive cells in SH-SY5Y neuroblastoma cells transfected with BRN2-specific siRNA for 48 h prior to treatment with 1 µM cisplatin for 24 h. Error bars indicate mean +/- S.D. of three biological replicates. Analysis by Paired Student's t-test. ** *P* = <0.01, *** P=<0.001. A western blot showing the decrease in BRN2 expression after siRNA transfection and before treatment with cisplatin is shown to the right.

Herbert et al Supplemental Figure S7

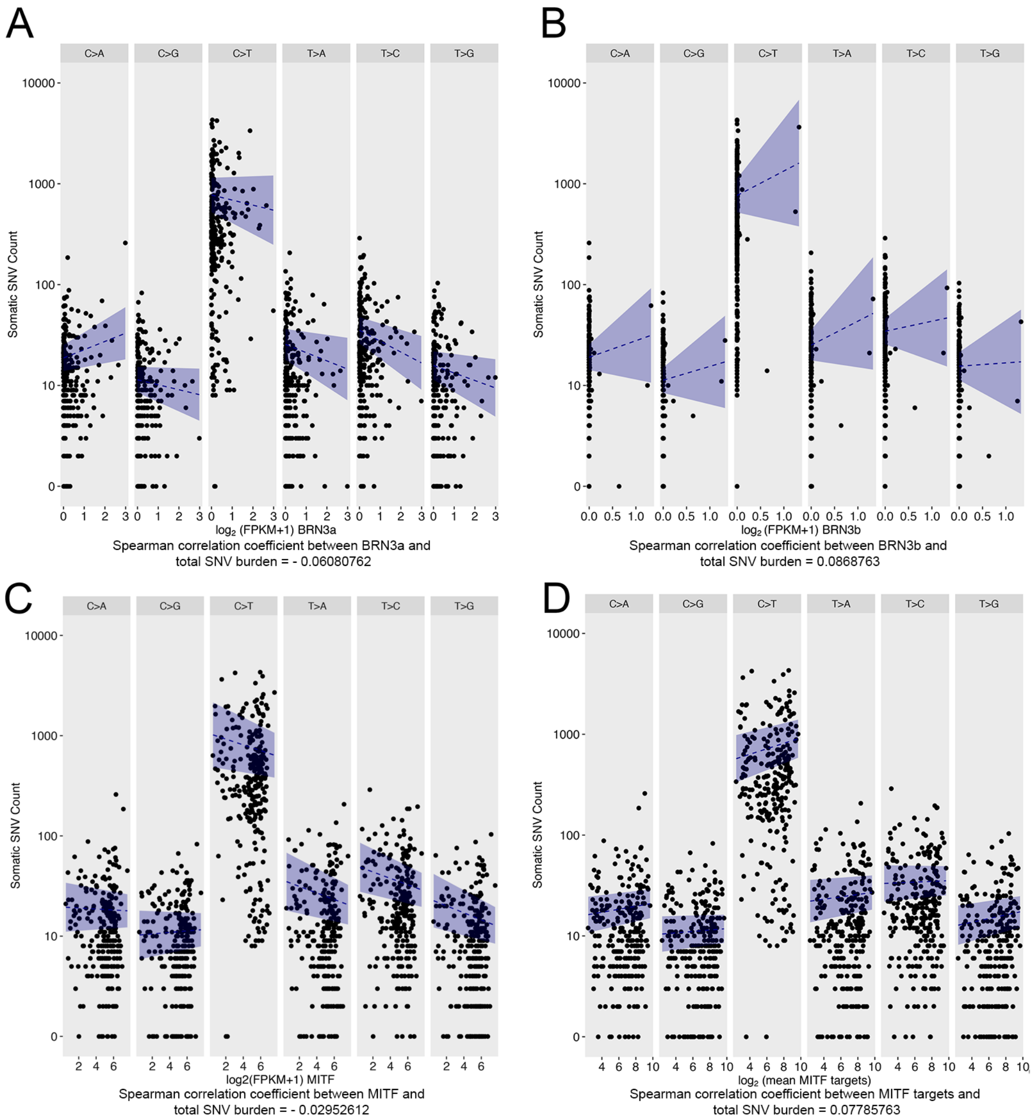

Figure S7. SNV counts were plotted against $\log_2$(FPKM+1) values for BRN3a (*A*), BRN3b (*B*), MITF (*C*), or the average expression of a well-characterised MITF target gene set (mean FPKM counts) (*D*). For each SNV class, the blue dashed line (and ribbon) charts the predicted mean mutation burden (and 95% confidence interval) of a patient with the most common constellation of values for clinical variables as the $\log_2$(FPKM+1) BRN3a, BRN3b, MITF, or MITF-target mean gene set expression value increases from the minimum to the maximum observed in the TCGA dataset, with all other clinical variables held fixed. In these models and for this prediction, the value for the BRN2 expression variable was set to 1.023459, the median $\log_2$ (FPKM +1). The MITF target gene set comprises *MITF, BCL2, CDK2, CDK4, DCT, HIF1A, MLANA, SILV, PPARGC1A, RAB27A, SHC4, TRPM1, TYR, TBX2, MCOLN1, TYRP2, DIAPH1, MET.*
